# Predictive modeling of clinical trial terminations using feature engineering and embedding learning

**DOI:** 10.1038/s41598-021-82840-x

**Published:** 2021-02-10

**Authors:** Magdalyn E. Elkin, Xingquan Zhu

**Affiliations:** Department of Computer & Electrical Engineering and Computer Science, Florida Atlantic University, Boca Raton, FL 33431 USA

**Keywords:** Data integration, Data mining, Data processing, Data publication and archiving, Databases, Literature mining, Machine learning, Predictive medicine, Software, Computational biology and bioinformatics, Medical research, Clinical trial design

## Abstract

In this study, we propose to use machine learning to understand terminated clinical trials. Our goal is to answer two fundamental questions: (1) what are common factors/markers associated to terminated clinical trials? and (2) how to accurately predict whether a clinical trial may be terminated or not? The answer to the first question provides effective ways to understand characteristics of terminated trials for stakeholders to better plan their trials; and the answer to the second question can direct estimate the chance of success of a clinical trial in order to minimize costs. By using 311,260 trials to build a testbed with 68,999 samples, we use feature engineering to create 640 features, reflecting clinical trial administration, eligibility, study information, criteria etc. Using feature ranking, a handful of features, such as trial eligibility, trial inclusion/exclusion criteria, sponsor types *etc.*, are found to be related to the clinical trial termination. By using sampling and ensemble learning, we achieve over 67% Balanced Accuracy and over 0.73 AUC (Area Under the Curve) scores to correctly predict clinical trial termination, indicating that machine learning can help achieve satisfactory prediction results for clinical trial study.

## Introduction

Clinical trials are studies aiming to determine the validity of an intervention, treatment, or test on human subjects. Randomised controlled trials, where participants are allocated at random (by chance alone) to receive one of several clinical interventions, are the ultimate evaluation of a healthcare intervention. Effective clinical trials are necessary for medical advancements in treating, diagnosing, and understanding diseases^1,2^. Since 2007, under the Food and Drug Administration Amendments Act (FDAAA), clinical trials are required to be registered to an online database (ClinicalTrials.gov) if they have one or more sites in the United States, conducted under an FDA investigational new drug/devise, or involve a drug/device product manufactured in the U.S. and exported for research. Trials requiring approval of drugs/devices are required to submit results within one year of completion^3^. While the mandate specifies type of trials legally required to submit results, majority of trials with results posted on the database are not legally obligated to do so^4^. The database currently lists 311,260 studies (as of May 2019).

The ClinicalTrials.gov database serves as a way to access summary and registration information for completed and terminated clinical studies, where terminated trials are those whose recruiting participants have stopped prematurely and will not resume and participants are no longer being examined or treated. There are many obstacles to conducting a clinical trial. Time frames, number of participants required, and administrative efforts have increased due to several factors: (1) an industry shift to chronic and degenerative disease research; (2) non-novel drug interventions requiring larger trials to identify statistical significance over the existing drug intervention; (3) increased complexity of clinical trial protocols; and (4) increased regulatory barriers^5^. These factors inflate the financial costs of clinical trials and increase the likelihood of a trial becoming terminated.

### Clinical trial terminations

Clinical terminations result in significant financial burden. Estimates of drug development are around 1.3 billion dollars and are rising at a rate of 7.4%, largely in part to clinical trial costs^5^. Terminated trials are associated with opportunity costs that could have been applied to other efforts^4^. Secondly, there are ethical and scientific issues surrounding terminated clinical trials. All subjects consenting to participate in a clinical trial do so to contribute to the advancement of medical knowledge. If a trial is terminated, subjects are not always informed about the decision and associated reasons^6^, resulting in direct loss of personal benefit from an interventional study^7^. Thirdly, terminated trials also represent a loss of scientific contribution to the community. Often relevant information about why a study was terminated is not reported and results and/or protocols are not published^8^.

To protect the health and safety of participants in a clinical trial, if data collected indicates negative side effects/adverse events, the trial will be terminated. Interventional trials often employ a data and safety monitoring committee that could recommend termination based on patient safety concerns^9^. Observational studies do not introduce an intervention in the participants, thus they are less likely to terminate due to safety concerns. The FDA states the preferred standard for clinical trial practice is to only terminate with clear evidence of harm from data within the study or as result of published findings from other studies^7^. In reality, this isn’t always the case. Often there are administrative issues such as logistical difficulties, loss of staff members, inadequate study design, protocol issues, etc.^4,10^, resulting in trial termination.

A terminated trial indicates that the trial already started recruiting participants but stopped prematurely and recruited participants are no longer being examined/treated^11^. Studies, using 8,000 trials, found that 10-12% of clinical trials are terminated^4,10,12^. Reasons include insufficient enrollment, scientific data from the trial, safety/efficacy concerns, administrative reasons, external information from a different study, lack of funding/resources and business/sponsor decision^4,8,10,12^.

Insufficient patient enrollment is often the greatest factor resulting in termination^4,10,12^. The ability to detect a significant effect is directly tied to the sample size. If the intended target enrollment is not met, the study’s intended effect will decrease due to less power^13^. Previously it was shown that eligibility criteria, non-industry sponsorship, earlier trial phase and fewer study centres are partially associated with insufficient enrollment^14^. Lack of funding has also been identified as a major reason for early termination^4,10^. Average costs of clinical trials range from 1.4 million up to 52.9 million^5^. It has also been shown that the number of publicly funded clinical trials have decreased from the years 2006-2014, while the number of industry funded clinical trials have increased^15^. However, industry sponsorship doesn’t guarantee that a clinical trial will be completed. There has been cases where a company can prematurely terminate a clinical trial due to commercial/business decisions^7,9^. Commercial decisions for an industry don’t necessarily represent a lack of funding, but a lack of perceived profit from continuing the pursuit of the intervention being studied in the clinical trial.

### Related work

A previous study modeled clinical trial terminations related to drug toxicity^16^, by integrating chemical and target based features to create a model to distinguish failed toxic drugs from successful drugs^16^. While drug toxicity is a common factor for clinical trial terminations, many clinical trials terminate due to other reasons^4, 10^.

Two previous studies utilized clinical trial study characteristics and descriptions from the ClinicalTrials.gov database to predict terminations^17,18^. The first study^17^ tokenizes the description field to find high/low frequency words in terminated/completed trials as features to train a binary predictive model. The second study^18^ uses Latent Dirichlet Allocation to find topics associated to terminated/completed trials. The corresponding topic probabilities are used as variables in predicting clinical trial terminations. Both studies determined that the addition of unstructured data to structured data increases the predictive power of a model for terminated clinical trials^17,18^. These results provide validity to our research design of using structured and unstructured information as variables to predict clinical trial terminations. Similar to the previous studies, we utilize study characteristics and description fields for variables in a model to predict clinical trial termination. However, our research differs in significant ways: (1) we design features to represent important information from the unstructured eligibility requirement field; (2) we include more study characteristic fields to represent administrative features of clinical trials; (3) we utilize the keywords field from the clinical trial report; and finally, (4) we use word-embedding to capture unstructured description fields. Using a word-embedding model, we are able to represent the whole description field as a numerical vector, without determining words or topics associated to completed or terminated trials to create features, for predictive modeling.

### Contribution

The goal of our study is to determine main factors related to terminated trials and to predict trials likely to be terminated. The main contribution of the study is as follows.

- Large scale clinical trial studies: Our research delivers a large scale clinical trial reports database for termination study. The database, including features and supporting documents, are published online to benefit the community^19^.
- New features: Our research creates a set of new features, including eligibility features and administrative features, to characterize and model clinical trials. In addition, our research is the first effort to explore using embedding features to model clinical trials. The results show that embedding features offer great power for prediction. Further more, the results indicate that the combination of statistic features, created from clinical trial structural information, keyword features and embedding features have the highest predictive performance.
- Predictive Modeling and Validation: Comparing to existing studies^17,18^, we investigate a variety of learning algorithms to address class imbalance and feature combinations for clinical trial termination prediction. Our model achieves over 0.73 AUC and 67% balanced accuracy scores for prediction, representing the best performance for open domain clinical trial prediction. The rigorous statistical tests provide trust-worth knowledge for future study and investigation.

## Methods and materials

### Clinical trial reports

A total of 311,260 clinical trials taking place in 194 countries/regions, in XML (Extensible Markup Language) format, were downloaded from ClinicalTrials.gov in May 2019. If a trial had sites in multiple countries, the country with the most sites is recorded. In the case of a tie, the first country listed for trial site is recorded. The top 25 countries are determined as those with at least 1,000 clinical trials. The top 10 of these countries are shown in Table 1(a) where 34% (106,930) trials are in the United States. The trials cover a wide range of research fields from diseases such as cancer, infectious diseases etc. to mental health conditions and public health and safety. Table 1(b) reports the top 10 clinical fields, based on MeSH (Medical Subject Headings) term frequencies in the trials. Supplementary Figure 2 lists inclusion criteria to build dataset for our study. From 311,260 trials, we select Completed or Terminated trials, starting in or after 2000, belonging to one of the top 25 countries, and having no missing values for the keyword and detailed description field. The final number of trials in the testbed was 68,999, where 88.54% (61,095) are completed and 11.46% (7,094) are terminated.

**Table 1:** County and research field statistics of the clinical trials used in the study.

Countries	# of Trials	Countries	# of Trials
**(a) Top-10 countries/regions**			
United States	106,930	U. K.	9,084
France	16,460	Korea (R)	7,355
Canada	15,558	Belgium	6,008
China	13,948	Australia	5,717
Germany	10,004	Italy	5,664

MeSH Terms	# of Trials	MeSH Terms	# of Trials
**(b) Top-10 research fields (DM stands for “diabetes mellitus”)**			
DM (Diabetes mellitus)	9,315	Carcinoma	4,930
Breast Neoplasms	7,049	Lung neoplasms	4,758
Syndrome	6,591	Leukemia	4,642
DM (Type 2)	5,781	HIV Infections	4,576
Disease	5,079	Depression	4,478

**Figure 1:**
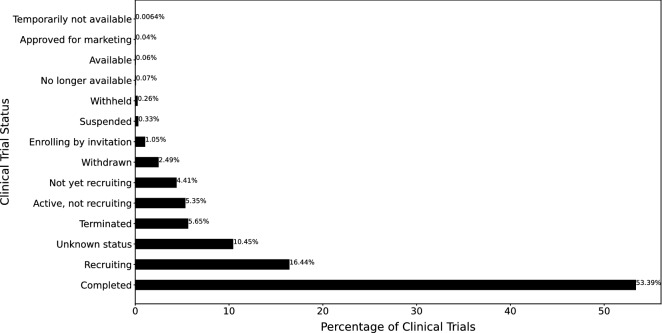
Summary of clinical trial statuses. The *x*-axis shows the % of clinical trials, and the *y*-axis shows the trial status.

The status field in the clinical trial report represents the recruitment status of the whole clinical study. The listed options for Status includes, “Not yet recruiting”, “Recruiting”, “Enrolling by invitation”, “Active, not recruiting”, “Completed”, “Suspended”, “Terminated”, and “Withdrawn”^11^. Overall, the first four indicate studies that are currently in progresses or will begin progress in the future. “Completed”, “Terminated”, and “Withdrawn” trials represent those which are completed or prematurely ended. For a trial to be “Withdrawn” it had to stop prior to enrolling it’s first participants. “Suspended” trials are those which have stopped early but may start again. For expanded access clinical trials, statuses could also include “Available”, “No longer available”, “Temporarily not available” and “Approved for Marketing”. “Unknown” indicates that the trial’s last known status was recruiting, not yet recruiting or active, not recruiting, however the trial passed it’s completion date and the status has not been verified within the last 2 years^11^. Figure 1 summarizes status of all 311,260 trials, where 53.3894% (166,180) are “Completed” and 5.6464% (17,575) are “Terminated”.

### Clinical trial feature engineering

In order to study factors associated to trial terminations, and also learn to predict whether a trial is likely going to be terminated or not, we create three types of features: statistics features, keyword features, and embedding features as follows.

#### Statistics features

Statistic features use statistics *w.r.t.* administrative, eligibility, study design, and study information to characterize trials.

##### Administrative features

**Figure 2:**
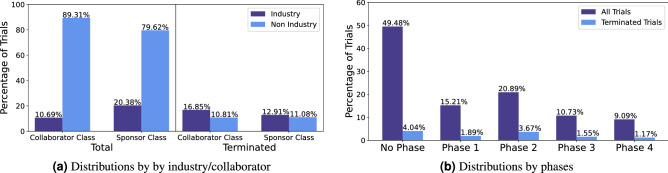
Distributions of clinical trials by collaborator and sponsor classes (**a**); and by trial phases (**b**).

include number of collaborators, number of officials, industry sponsorship, industry collaborator and the type of responsible party. Previously it was shown that 9.4% of clinical trials terminate prematurely due to trial administration or conduct^4^. The number of collaborators and officials for a clinical trial affect the management of the trial. Clinical trial officials are those responsible for the scientific leadership of the protocol. Collaborators are organizations other than the sponsor that provide support for a clinical study. Support may include funding design, implementation, data analysis or reporting^11^. For clinical trials, the sponsor and collaborator class include “Industry”, “NIH”, “U.S.Fed”, and “Other”. Industry sponsorship/collaborator have several different potential factors for termination. As discussed in previous sections, industry sponsors may have more funding but can terminate due to business decisions. An industry collaborator may provide key funding/regulatory assistance for a non-industry sponsored clinical trial. Of all 68,999 final selected trials, 20.38% (14,064) had industry sponsorship. For non-industry sponsored clinical trials, 11.08% (6,088) were terminated, compared to 12.91% (1,816) terminated trials for industry sponsorship. For collaborators, if there were more than one collaborator, the most common collaborator class was recorded, and in the case of a tie, the first collaborator class listed was recorded. In total, 10.69% (7,379) clinical trials main collaborator class was industry. For non-industry collaborator clinical trials, 10.81% (6,661) were terminated, compared to 16.85% (1,243) terminated trials with industry collaborator. The difference between industry/non industry is evidently higher when looking at the collaborator class compared to the sponsor class. The distributions of percentage of terminated trials for sponsor and collaborator class are shown in Fig. 2a.

##### Study information features

intend to describe basic information about the clinical trial. These features include if the clinical trial has expanded access, Data Monitoring Committee (DMC) regulation, FDA regulation, study type (international or observational), the phase of the trial, and if the study was in USA or outside USA. A trial with expanded access provides participants with serious health conditions or diseases access to medical treatments that are not yet approved by the FDA. The FDA regulations state that clinical trials with expanded access can transition to an investigational new drug (IND) protocol. An IND protocol is necessary to provide evidence for FDA approval. If a clinical trial with expanded access wants to transition to an IND protocol, the trial with expanded access protocol will be terminated^20^. DMC regulation indicates that the clinical trial has a data monitoring committee, groups of independent scientists monitoring the safety of participants, for the study. The DMC committee is responsible to provide recommendations regarding stopping the trial early for safety concerns.

Phases of clinical trials include: No phase, early phase 1, phase 1/2, phase 2, phase 2/3, phase 3, or phase 4. No Phase are trials without defined phases, such as in studies of devices or behavioral interventions. Early phase 1 are exploratory trials involving minimal human exposure with no diagnostic intent, these include screening studies and micro-dosing studies. Phase 1 are trials with initial studies to determine the metabolism and pharmacologic action of drugs in humans. These aim to uncover any side effects with increasing doses and early evidence of effectiveness. Phase 1/2 trials are combinations of phase 1 and phase 2. Phase 2 trials are controlled clinical studies to evaluate the effectiveness of the drug for a particular indication. These trials include participants with the disease or condition under study and the trial aims to determine the short term side effects and risks. Phase 2/3 trials are combinations of phase 2 and phase 3. Phase 3 trials determine the overall benefit-risk relationship of the drug. Phase 4 trials are studies of FDA-approved drugs to determine additional information of the drugs risk, benefits and optimal usage^11^. The motivation for using the trial’s phase was to determine if phase was related to termination. A previous study that looked at termination reasons found that early phase trials are more likely to terminate due to scientific reasons while later phase trials have more complicated reasons for termination^10^. While phase alone is not an indicator of trial terminating, it might be likely that the combination of phase and another feature can indicate that a clinical trial will be terminated. The distribution of clinical trials by phase is shown in Fig. 2b.

**Figure 3:**
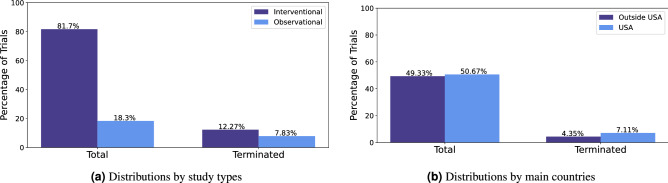
Distributions of clinical trials by study types (**a**); and by main countries (**b**).

Interventional studies introduce a treatment plan for participants, such as drugs, vaccines, surgery, devices or non-invasive treatments such as behavioral changes or education. Observational studies do not introduce treatment plans, participants are observed for health outcomes^11^. The majority of the clinical trials used for analysis, 81.7% (56,369) are interventional studies, 18.3% (12,630) are observational studies. This is mostly likely due the fact that observational studies are often not registered. Moreover some observational studies are registered after publication^21^. Interventional studies have a higher rate of termination, 12.12% (6,915) interventional studies were terminated compared to 7.83% (989) observational studies were terminated. The distribution of interventional and observational studies is shown in Fig. 3a.

Clinical trials could have sites located in different countries/regions. A clinical trial’s main country was determined by the country with the largest number of sites for the clinical trial. Majority 50.6% (34,964) of clinical trials’ main country was USA. Accordingly, we create a binary feature indicating if the clinical trial main country was USA or outside of USA. Although the FDA regulations for trials to register in the ClinicalTrials.Gov database mainly applies to clinical trials in the USA, many international trials register to the database as well. The International Committee of Medical Journal Editors (ICMJE) issued a clinical trial registration policy as part of the ICMJE recommendations for conduct, reporting, editing and publication of scholarly work in medical journals. The recommendations encourages journal editors to require clinical trials registered before the start of a study that is considered for publication. The World Health Organization (WHO) also instituted a policy, the International Clinical Trials Registry Platform (ICTRP) that specifies the registration of all interventional trials is a scientific, ethical and moral responsibility^22^. Therefore, many international studies register their trials in the ClinicalTrials.gov database to meet the requirements for publication in some journals and to adhere the policies of the WHO. The motivation to using USA/non-USA as a feature is to capture any differences between trials inside the United States and outside the United States. Clinical trials in USA had a higher rate of termination with 7.11% (4,905) trials terminated. The distribution of outside USA vs. USA clinical trials and termination is shown in Fig. 3b.

##### Study design features

**Figure 4:**
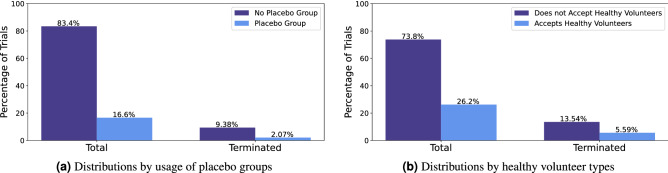
Distributions of clinical trials by placebo groups (**a**); and by volunteer types (**b**).

focus on study design of a clinical trial, which plays an important role in the success/termination of a trial. The study design features include the number of groups, number of countries, number of sites, whether the clinical trial has randomized groups, the masking technique for groups, and whether the study included a placebo group. Adding randomized groups and the masking technique for groups introduces logistical difficulties in a clinical trial study. More complicated protocols introduce complex issues that may lead to early termination. More groups needed for a clinical trial indicate more higher required patient enrollment, if this is not met, the trial will have to terminate. Likewise if a study has fewer sites, the number of required patients might not be found. It was previously shown that studies with fewer study sites are more likely to not reach target patient enrollment^14^. Thus if a clinical trial has fewer sites, it might not reach patient enrollment and terminate. However, increasing the sites for a clinical trial increases the resources (funds/personnel) required for monitoring each site. Although the use of a placebo group is often required for a clinical trial, it was shown that trials with placebo groups are a risk factor for insufficient patient enrollment^14^. The addition of a placebo group indicates that the trial needs higher numbers of participants. If this is not met, the trial will suffer from insufficient patient enrollment and be terminated. The distribution of placebo groups is shown in Fig. 4a.

##### Eligibility features

capture information about eligibility requirements in clinical trials. As discussed in previous sections, eligibility is often a key factor in trial termination. We used basic eligibility fields from the clinical trial reports (if eligibility requirement is present, gender restriction, age restriction, acceptance of healthy volunteers) and created features from the eligibility field text block to encapsulate key points about the eligibility requirements. The eligibility criteria can be separated into inclusion criteria or exclusion criteria. Some trials do not indicate a clear separation of inclusion criteria or exclusion criteria, so the total eligibility field was considered as well. The eligibility criteria field can be separated into the number of criteria per inclusion/exclusion/total eligibility by the number of lines per inclusion/exclusion/total eligibility. The number of criteria was considered, the average number of words per line and the total number of words per inclusion/exclusion/total eligibility were all created as features. The number of numeric numbers was also considered for inclusion/exclusion/total eligibility was considered as well. The larger number of lines per eligibility indicate more strict requirements. A larger number of words also indicates higher requirements for eligibility. A trial with a high number of numeric values indicates the trial has very specific eligibility requirements (such as age, metabolic levels, ability to withstand a certain dosage, etc.). The majority of trials, 73.8% (50,920), did not accept healthy volunteers. Trials not accepting healthy volunteers had a higher rate of termination, 13.54% of trials (6,893). The distribution of clinical trials by acceptance of healthy volunteers is shown in Fig. 4b.

### Keyword features

The detailed description field in the clinical trial report is an extended description of the trial’s protocol. It includes technical information but not the entire study’s protocol. The keyword field is words or phrases to best describe the study’s protocol. They are used to help users find studies when querying the online database^11^. Keywords are created by the clinical trial register using the US National Library of Medicine (NLM) Medical Subject Heading (MeSH) controlled vocabulary terms. MeSH was developed by NLM to properly index biomedical articles in MEDLINE^23^.

The motivation of using keyword features is to represent the clinical trial’s research area as determined by keywords. To create features capturing information about keywords, TF-IDF (term frequency-inverse document frequency) was used, where TF is the frequency of the term in the document and IDF is measure of term specificity, based on counting the number of documents that contain the term. The concept of IDF is that a term that occurs in many documents, such as the term “the”, is not a good discriminator. These terms are given less weight than ones that occur in a few documents^24^. TF-IDF is used to measure the importance of a keyword compared to all keywords in the clinical trial reports. Keywords in clinical trial documents are composed of multiple MeSH terms. For example, if a clinical trial has two listed keywords, “Ankle Joint” and “Osteoarthritis”, then the resulting document has three keywords: “Ankle”, “Joint” and “Osteoarthritis”. Keywords are extracted from the keyword field by tokenizing the field, separated with punctuation and spaces, and stop words are removed. After finding the TF$\text{- }$IDF(*f*) value for each keyword *f*, using all (68,999) clinical trials, the top 500 terms are used as keyword features. The top 20 keyword features as determined by their TF-IDF score is shown in Table 2 (a). For each trial, the resulting TF-IDF score for each keyword is used as input to the classification models.

### Embedding features

The keyword features in the above subsection only provide word level information about clinical studies. A common dilemma is that the number of keyword features should be relatively large, in order to capture specific information of individual trials. As the number of keyword feature increases, the feature space will become sparse (with many zeros), because some keywords only appear in a small number of studies. In order to tackle this dilemma, we propose to create embedding features, which will generate a dense vector to represent detailed descriptions of each clinical trial report. Two distinct advantage of the embedding features is that (1) we can easily control the embedding feature size to be a relatively small number (typical 100 or 200), and (2) the embedding feature space has dense feature values normalized in the same range.

To represent the detailed description field as a vector input into the classifier, Doc2Vec was used. Doc2Vec^25^ is an expansion of Word2Vec^26^, a neural network to generate vector representations of words^27^. In the continuous bag-of-words (CBOW) implementation of Word2Vec, a word is predicted by the words in the surrounding context. Context words are used to predict the current word^25^. For example, given a training sentences, such as “autologous stem cell transplantation”, Word2Vec will use the co-occurrence of words to train word embedding models. Because “stem” and “cell” both occur in the sentence, it will then set input corresponding to “stem” as one, and expect the output nodes corresponding to “cell” to have the largest output. Every word in the sentence is mapped to a unique vector in a column of matrix *W*. These vectors are concatenated or averaged together to predict the next word in the sentence. The result creates vector representations of words where similar words will have similar vector representations. For example, “Patient” will have a similar vector to “Subject”, and “Physician” will have a similar vector to “Doctor”, as shown in Table 2(b) and (c).

By using a neural network model similar to Word2Vec, Doc2Vec^25^ adds each each document as an extra input (in addition to the words). After training the model using all clinical trial documents, the *d* dimensional weight values connecting each document to the neural network will be used as the embedding features to represent each document. The Doc2Vec model creates a vector of length 100 to represent the detailed description. The vector is ultimately used as 100 different features for our final predictive models.

**Table 2:** Top 20 keywords with the largest TF-IDF scores (a), and the top 10 words and their cosine similarities to “Patient” (b) and “Doctor” (c) determined by using trained Doc2Vec word embedding vector.

(a) Top 20 keywords in TF-IDF scores	
Keyword feature	
Verrucous	Larynx
Testicular	Lip
Nasopharynx	Noncontiguous
Paranasal	Endometrial
Contiguous	Esophagus
Salivary	Sinus
Neuroblastoma	Astrocytoma
Hypopharynx	Uterine
Gland	Migraine
Sezary	Cleaved

(b) Top 10 words to “Patient”	
Word	Similarity
Subject	0.933080
Participant	0.920826
Infant	0.756492
Woman	0.747329
Child	0.725826
Neonate	0.725279
Volunteer	0.670207
Person	0.662123
Mother	0.659572
User	0.642564

(c) Top 10 words to “Doctor”	
Word	Similarity
Physician	0.780111
Coordinator	0.747168
Clinician	0.709273
Staff	0.696269
Psychiatrist	0.684473
Oncologist	0.661595
Surgeon	0.638823
Physiotherapist	0.637646
Sponsor	0.634423
Investigator	0.634007

### Termination key factor discovery

The feature engineering approaches in the above subsections will create a set of potential useful features (or key factors) associated to the clinical trial termination. In order to determine features playing important roles to the trial termination, we will use *feature selection* to rank all features, based on their relevance to the class label (*i.e.* trial termination). Three types of feature selection approaches, filter, wrapper, and embedded method^28^, are commonly used for feature selection. In our research, since we are interested in single features most relevant to the target class, independent of any learning algorithms, we use filter approaches to rank all features, according to their relevance scores to the class label. Five feature selection methods, including ANOVA (Analysis of Variance), ReliefF, Mutual Information (MI), CIFE (Conditional Informative Feature Extraction) and ICAP (Interaction Capping), are used in the study.

Due to the inherent difference of the feature evaluation mechanism, feature selection methods assess feature importance from different perspectives, resulting in different orders of feature importance. To combine their feature ranking results, we employ Dowdall Aggregation (DA) to aggregate feature rank from all methods. Dowdall system is a variant of Borda count which assigns a fraction number, inverse to the ranking order of each feature, as the weight value for each ranking method. Overall, Dowdall method favors features with many first preferences (top ranking candidates). If a feature $f_i$ is accidentally ranked to the bottom of the feature list by a method, it will have very little impact to $f_i$’s DA aggregation value because it contributes a small fraction weight values to the final aggregation.

### Clinical trial termination prediction

In order to predict whether a clinical trial may be terminated or not, we use features created from the above steps to represent a clinical trial, and train four types of classifiers, Neural Networks, Random Forest, XGBoost, and Logistic Regression to classify each trial into two categories: “Completed” *vs*. “Terminated”. The final data set used for analysis has 88.54% completed trials (61,095) and 11.46% terminated trials (7,094), meaning the ratio between terminated *vs.* completed trials is 1 to 7.75. A class imbalance problem occurs when there are many more instances of one class compared to another. In these cases, classifiers are overwhelmed by the majority class and tend to ignore minority class samples^29^. Accordingly, we employ random under sampling to handle the class imbalance problem, which is widely accepted for handling class-imbalance^29^.

#### Random under sampling

takes samples from the majority class to use for training along with the instances of the minority class. In this study, random under sampling is applied to the majority class to produce a sampled set with an even number of majority class and minority class samples. Prior to random under sampling, the imbalanced ratio of terminated trials to completed trials is 1 to 7.75. After random under sampling, the imbalanced ratio of terminated trials to completed trials is 1 to 1. Because random under sampling may potentially remove important examples and result in bias in trained models^29^. We repeat random under sampling 10 times, each time procures one sampled data set trains one model. The 10 trained models are combined (using ensemble) to predict test samples.

Supplement includes the clinical trial prediction framework details and comparisons between different sampling ratios.

## Results

### Experimental settings and performance metrics

We use five fold cross-validation in our experiments, all models are tested on an unique hold out test set of 20% (13,780) trials, for five times, to evaluate their performance. After the validation sets are created, Doc2Vec is trained on each training data set and the Doc2Vec model infers a vector for the “Detailed Description” field for each separate training and test data set. Supplement includes details on the Doc2Vec implementation.

Four different classification models, Neural Network, Random Forest, Logistic Regression and XGBoost, are comparatively studied. The Neural network model consists of a multi-layer network with 1 hidden layer and 100 nodes, and Random Forest consists of 1,000 fully grown trees. The Supplement provides additional information about model hyperparameters. To optimize parameters, randomized grid search was initially used to narrow parameter values; followed with exhaustive grid search to determine final optimal parameters. To determine the results from feature engineering, single models are tested with statistics features only, keyword features only, word embedding features only and then combinations of the three. To determine the overall prediction results, all features are used with a single model method and with ensemble model method, respectively.

Four types of performance measures, accuracy, balanced accuracy, F1-score, and AUC values are reported in the experiments. Supplement provides additional details about each measure.

### Termination key factor detection results

Using feature engineering approaches, we design 40 statistics features, 500 keyword features, and 100 embedding features. In order to understand their importance for trial prediction, we report the aggregated feature ranking (using Dowdall Aggregation) in Table 3, where a superscript ($^{{s, k, e}}$) denote a statistics feature, a keyword feature, and an embedding feature, respectively. The value in the parenthesis denotes Dowdall ranking. For example, “Eligibility Words$^s$ (2)” denotes that this is a statistics feature and is ranked no. 2 out of all 640 features. The left most column show the top 20 statistics features in the left most column. The middle column shows the top 20 keyword features and their their respective ranking. The right column shows the top 20 ranked features out of all features. Embedding features belong to a vector of size 100 from the vector representation of the detailed description field. The feature names for embedding features represent their index position in the vector, {0:99}. The top ranked feature, 8, is the 9th index position of the detailed description document vector. Table 1 in the Supplement further lists the top 40 Statistics Features, Keyword Features and overall ranked features.

Overall, statistics features about eligibility are ranked high, such as Eligibility words, no eligibility requirement, Inclusion criteria words, eligibility lines, average inclusion words per line, average eligibility words, etc. Half of the 40 top ranked features are statistics features, indicating logistics, study information, clinical designs, and eligibility are crucial to trial completion or termination. Keyword features provide information about the research or therapeutic area of the clinical trial. Out of the top 10 keyword features, all are cancer related except for “Germ”. Within the oncology related terms, the keywords “Mycosis”, “Fungoides”, and “Sezary” are all interrelated and in the top 10 ranked keyword features. Mycosis fungoides and Sézary syndrome are types of Cutaneous T-cell lymphomas, which are rare diseases affecting 10.2 per million people^30^.

**Table 3:** The top 20 Statistics Features (1${st}$ column), Keyword Features (2${nd}$ column), and overall ranked features (3${rd}$ column) using Dowdall Aggregation. The superscripts $(^s, ^k, ^e)$ denote feature types (statistics features, keyword features, or word embedding features, respectively). The number in the parenthesis denotes the aggregated ranking of the feature, with (1) being the best ranking. Table 1 in the Supplement further lists the top 40 features.

Statistics Features	Keyword Features	All Features
Eligibility Words$^s$ (2)	Verrucous$^k$ (6)	8$^e$ (1)
No Eligibility Requirement$^s$ (3)	Testicular$^k$ (8)	Eligibility Words$^s$ (2)
Inclusion Words$^s$ (5)	Neuroblastoma$^k$ (13)	No Eligibility Requirement$^s$ (3)
Number Countries$^s$ (7)	Sezary$^k$ (20)	1$^e$ (4)
Phase 1$^s$ (10)	Fungoides$^k$ (27)	Inclusion Words$^s$ (5)
Eligibility Lines$^s$ (11)	Nasopharynx$^k$ (31)	Verrucous$^k$ (6)
Number Arms$^s$ (12)	Mycosis$^k$ (32)	Number Countries$^s$ (7)
Industry Sponsor$^s$ (14)	Contiguous$^k$ (33)	Testicular$^k$ (8)
Average Inclusion Words$^s$ (15)	Germ$^k$ (39)	13$^e$ (9)
Average Eligibility Words$^s$ (17)	Thyroid$^k$ (42)	Phase 1$^s$ (10)
Exclusion Words$^s$ (18)	Noncontiguous$^k$ (48)	Eligibility Lines$^s$ (11)
Number Officials$^s$ (19)	Paranasal$^k$ (50)	Number Arms$^s$ (12)
Average Exclusion Words$^s$ (21)	Myelomonocytic$^k$ (51)	Neuroblastoma$^k$ (13)
Random Groups$^s$ (22)	Hypopharynx$^k$ (57)	Industry Sponsor$^s$ (14)
Eligibility Numbers$^s$ (24)	Uterine$^k$ (60)	Average Inclusion Words$^s$ (15)
Inclusion Lines$^s$ (25)	NSCLC$^k$ (61)	16$^e$ (16)
Exclusion Lines$^s$ (26)	Oropharynx$^k$ (63)	Average Eligibility Words$^s$ (17)
Healthy Volunteer$^s$ (28)	AML$^k$ (71)	Exclusion Words$^s$ (18)
Exclusion numbers$^s$ (34)	Salivary$^k$ (73)	Number Officials$^s$ (19)
Responsible Party: Sponsor$^s$ (37)	Remission$^k$ (74)	Sezary$^k$ (20)

### Feature engineering and combination results

**Figure 5:**
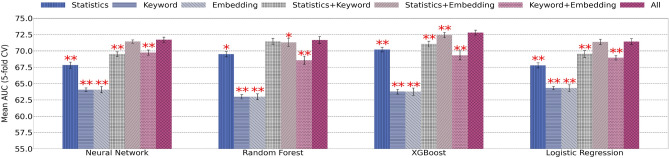
AUC scores for classifiers tested using different feature combinations. Each bar denotes clinical trial prediction result (AUC score) using one type of feature engineering method (or combination). Each group of bars (cluster) denote performance of one type of classifier. A single red star above a bar indicates a statistical difference with $p < 0.05$, two red stars indicate $p < 0.01$, compared to the models trained using all features.

In order to understand which type of features (or their combinations) are mostly informative for clinical trial termination prediction, we use different type of features (statistics features, keyword features, and word embedding features) and their combinations to train the four classifiers using a single model. The resulting AUC scores are reported in Fig. 5. For all models, the combination of all features demonstrates the highest performance. To verify the statistical difference, we performed a corrected resampled *t*-test, comparing results from all features to all other combinations, with respect to each model. Utilizing the Holm-Bonferroni corrected *p*-values, it was confirmed that using all features is significantly better than all other combinations except for Statististics+Embedding for Neural Network; Statistics+Keyword for Random Forest, and Keyword+Embedding for Logistic Regression.

Overall, the feature engineering results can be summarized into two major findings (1) for each type of features, statistics features have the best performance. Keyword and word embedding features have similar performance; (2) combining different types of features result in better classification results than using any single type of features alone, and using all features result in best classification results. Feature selection results in the Supplement (Figure 2) also confirm advantageous of using all features.

### Clinical trial termination prediction results

Table 4 reports the clinical trial termination prediction results, with respect to Accuracy, Balanced Accuracy, F1-score, and AUC scores. Because the dataset is severely imbalanced with 88.54% completed trials and 11.46% terminated trials, Accuracy scores are not reliable measures to asses classifier performance. Using a corrected re-sampled *t*-test^31^, comparing an ensemble model *vs.* its single model counterpart, the results show: All models have a significant increase in Balanced Accuracy and F1-score; all models are significantly different in accuracy; Random Forest shows a significant increase in AUC scores.

Ensemble XGBoost shows the highest scores in AUC and Balanced Accuracy, when using all features, compared to other Ensemble models. Using a corrected resampled *t*-test and Holm-Bonferroni corrected *p*-values, it was confirmed that XGBoost is significantly better, ($p < 0.01$), than Neural Network and Logistic Regression, which regards to AUC. XGBoost is slightly significantly better than Random Forest with $p = 0.056$. With regards to Balanced Accuracy, XGBoost is significantly better than all models with $p < 0.01$.

**Figure 6:**
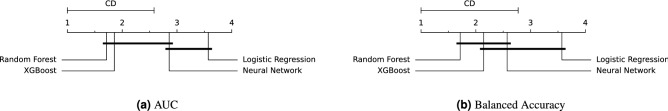
Critical difference diagram for ensemble models by comparing the four classifiers on different combinations of features; (**a**) AUC scores with $\alpha = 0.1$, the critical difference is 1.58; (**b**) Balanced Accuracy with $\alpha =0.5$, the critical difference is 1.77. Groups of classifiers that are not significantly different are connected.

To test the ensemble models performance over all combinations of features, a Friedman test shows a significant difference between the four ensemble models AUC scores, $\chi ^{2}_{F} = 9.686$, $p=0.021$. The Nemenyi post-hoc test, using $\alpha = 0.1$, results in Fig. 6a demonstrate that Random Forest and XGBoost are significantly better than Logistic Regression in AUC (There is no significant difference between Neural Network and the other three models in AUC). A Friedman test shows a significant difference between the four ensemble models in Balanced Accuracy, $\chi ^{2}_{F} = 7.971$, $p=0.047$. The Nemenyi post-hoc test, using $\alpha = 0.05$, results in Fig. 6b demonstrate that Random Forest is significantly better than Logistic Regression in Balanced Accuracy (There is no significant difference between Neural Network, XGBoost and the other three models in Balanced Accuracy). The Supplement lists results from all statistical tests. These statistical tests conclude that while XGBoost has highest performance with regards to using all features, Random Forest had reliable strength with regards to all feature combinations.

Overall, the results can be summarized into three major findings (1) ensemble model is always better (or much better) than single model in Balanced Accuracy, F1-score and AUC values; (2) single model learned from original dataset (without random under sampling) is not reliable (a classification model with several percent of F1-score typically means that one type of samples are largely misclassified); and (3) using random under sampling, ensemble model, and XGBoost result in the best trial termination prediction with over 0.73 AUC values and 67% Balanced Accuracy.

**Table 4:** Clinical trial termination classification results, using single model without random under sampling (a), and random under sampling based ensemble model (b) trained using all features. A * indicates where the ensemble classifier is significantly different from its single model classifier counterpart at $p<0.05$, and ** indicates a higher level of confidence at $p<0.001$.

(a) Single model termination classification				
Model	Accuracy	Balanced	F1-Score	AUC
Neural Network	88.47%	50.23%	1.21%	71.71%
Random Forest	88.54%	50.02%	0.10%	71.67%
XGBoost	88.55%	50.26%	1.18%	72.81%
Logistic Reg.	88.46%	50.48%	2.34%	71.42%

(b) Ensemble model termination classification				
Model	Accuracy	Balanced	F1-Score	AUC
Neural Network	62.66%**	66.42%**	30.43%**	72.03%
Random Forest	66.33%**	66.58%**	31.28%**	72.59%*
XGBoost	63.92%**	67.20%**	31.21%**	73.01%
Logistic Reg.	63.31%**	65.79%**	30.11%**	71.46%

## Discussion

Our study has twofold goals: (1) determine clinical trial termination key factors and (2) accurately predict trial termination.

For the first goal, among all studied features, statistics features are advantageous in describing tangible aspects of a clinical trial, such as eligibility requirements or trial phase. Some embedding features are ranked high, but the downside of embedding features is that the meaning of the detailed description field is not directly known, as it is represented as a numerical vector.

The top ranked keyword features indicate research areas more likely to be terminated. Our research shows that a majority of top ranked keyword features are cancer related. A previous study utilizing trial description field keywords also found oncology related terms such as “tumor”, “chemotherapy”, and “cancer” to be important keyword indicators^17^. The high ranking of oncology terms indicate that cancer trials pose a higher termination risk. Indeed, proving clinical effectiveness of therapeutic interventions in cancer has become increasingly complex. Although there is an increase in the number of cancer clinical trials, patient enrollment has, in fact, decreased^32^. Meanwhile, statistics features provide information on aspects of trials related to termination, and keyword features provide additional information on research areas susceptible to the factors identified by statistics features. For example, the high ranking of keywords, “Mycosis”, “Fungoides”, and “Sezary”, which are related to rare diseases, suggest that these trials may have troubles enrolling patients to meet eligibility criteria, ending in termination.

For the second goal, our research found that the combination of all features has the highest performance for all models. These results are in agreement with previous studies that use unstructured variables combined with structured variables (statistic features) for clinical trial termination models^17,18^. Our research, combined with existing findings, suggest that clinical trial termination is the outcome of many complex factors. High accuracy trial termination prediction should rely on advanced feature engineering approaches, instead of being limited to feature selection skills.

While previous studies^17,18^ only used Random Forest, our research demonstrates the predictive capabilities of other models: (1) Random Forest and XGBoost are superior to Logistic Regression when comparing performance over different combinations of features; (2) XGBoost is statistically superior to all models when considering performance with regards to all features; and (3) our ensemble methods are able to properly handle the class imbalance issue, which are very common in this domain.

Our research heavily relies on statistical tests. The Friedman statistical tests and critical difference diagrams demonstrate the classifiers rankings over different feature combinations. Because we used cross validation to find best parameters for each models, often their AUC scores for a specific feature combination were similar with a minor difference, which still impact their rankings, and directly affect their Nemenyi post-hoc tests. Unlike the corrected resampled *t*-test, the Friedman test and Nemenyi post-hoc tests do not take variability into overlapping training and test sets into account. The corrected re-sampled *t*-test can be more reliable with respect to pairwise comparison of one models performance to another. The Freidman tests demonstrate model superiority over all combinations of features.

## Conclusions

In this paper, we used feature engineering and predictive modeling to study key factors associated to clinical trial termination, and proposed a framework to predict trial termination. By using 311,260 clinical trials to build a dataset with 68,999 samples, we achieved over 0.73 AUC and over 67% Balanced Accuracy scores for trial termination prediction. The predictive modeling offers insight for stakeholders to better plan clinical trials to avoid waste and ensure success.

A limitation of our research is that the decision logic of the predictive models is not transparent, making it difficult to interpret the predictions. Future work can focus on models with better interpretability. In addition, research can segregate clinical trials into separate groups to determine if concentrated research area trials have more pronounced features or termination results. For example, this study and a previous study found oncology keywords as important features^17^. A different study has found surgery words as the highest important keyword factor^18^. Segregating clinical trials on the basis of research or therapeutic area for a single data set may possibly yield improved results for a predictive termination model. In which case, the same methodology could be applied to a subset of clinical trials.

## Supplementary Information

Supplementary Information.
