# Supplementary material for: Predictive modeling of clinical trial terminations using feature engineering and embedding learning
Source: Sci Rep. 2021 Feb 10;11:3446. doi: 10.1038/s41598-021-82840-x (PMC7876037; doi:10.1038/s41598-021-82840-x)
Supplement: Supplementary file 1 — Supplementary Information. [file 41598_2021_82840_MOESM1_ESM.pdf]

Supplement: Predictive Modeling of Clinical Trial Terminations using Feature Engineering and Embedding Learning

Magdalyn E. Elkin[1] **and Xingquan Zhu**[1,*]

[1]Dept. of Computer & Elecl. Eng. and Computer Science, Florida Atlantic University, Boca Raton, FL 33431, USA
[*]corresponding author: xzhu3@fau.edu

Supplement Information

Keyword Features

The Keywords field in a clinical trial has multiple MeSH terms. These are separated into single keywords, f, by tokenizing the field, separated with punctuation and spaces. Stop words, taken from the Natural Language Tool Kit (NLTK) package[1], are removed.

TF-IDF: Term Frequency-Inverse Document Frequency: TF-IDF of a keyword, f, in a clinical trial report T $\textit{tf-idf}(f,T)$, is computed as follows

$$\textit{tf-idf}(f,T) = \textit{tf}(f,T) \times \textit{idf}(f) \tag{1}$$

Where $\textit{tf}(f,T)$ is the number of times the term appeared in the keyword field in the clinical trial report T. This is multiplied by the IDF component, $\textit{idf}(f)$ of the term which is defined as

$$\textit{idf}(f) = \log\frac{1+n}{1+\textit{df}(f)} + 1 \tag{2}$$

Where n is the number of clinical trial reports, (n=68,999 in our experiments), and $\textit{df}(f)$ is the number of clinical trial reports that contained the term f in the keyword field. The resulting $\textit{tf-idf}(f,T)$ are then normalized by the Euclidean norm. After finding the $\textit{tf-idf}(f,T)$ values for each term f, (computed using TF-IDF Vectorizer implemented by the scikit-learn package for Python[2]), the top 500 terms are used as keyword features.

Embedding Features

Word2Vec Embedding Features: Formally, given a set of document $\mathscr{D}$ with m unique keyword: f_1, f_2, f_3,$\cdots$,f_m, continuous bag of word (CBOW)[3] based Word2Vec model aims to maximize the average log probability in Eq. (3), which is equivalent to predicting the appearance of a word f_i given observed b words, $f_{i-b+1} : f_{i-1} = \{f_{i-b+1}, f_{i-b}, \cdots, f_{i-1}\}$ within a context (such as a sentence or a paragraph).

$$\mathscr{L} = \sum_{i=1}^{\mathscr{D}} \log \mathbb{P}(f_i | f_{i-b+1} : f_{i-1}) \tag{3}$$

An example of the Word2Vec neural network model is showing in Figure 1. The input layer denotes m words (which correspond to the vocabulary of all documents). The hidden layer includes d neurons, which corresponds to the embedding feature size. The output layer also has m output nodes, each corresponds to a word in the input space. After training this network, each word in the input space will have d weight values connecting to the hidden layer. The d dimensional weigh values will be used as the embedding features to represent each word.

Doc2Vec Implementation: Doc2Vec is implemented using Gensim package for Python[4], utilizing Distributed Memory Model of Paragraph Vectors (PV-DM) which is analogous to the CBOW implementation of Word2Vec. The initial learning rate, α, is set as 0.05, and the training lasts 100 epochs. Minimum count is set as 5, meaning that words with frequency lower than 5 are ignored. Negative sampling is set to 5. The embedding vector size is fixed at 100, meaning Doc2Vec outputs a vector of length 100 to represent each clinical trial.

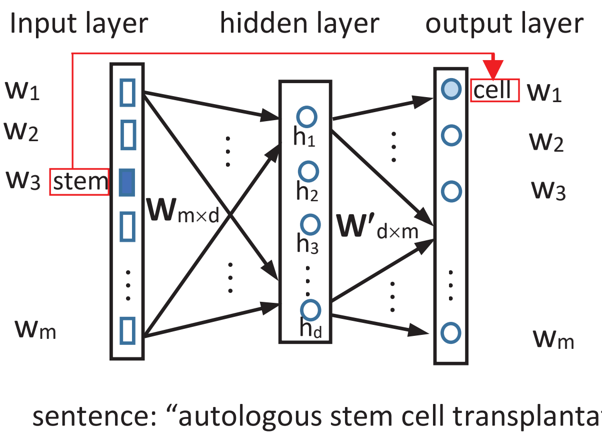

Figure 1. Word2Vec neural network architecture for word embedding learning. The input and output layer has the same dimension, which corresponds to the vocabulary of the document. The hidden layer has d nodes, which determines the embedding feature size. The principle of embedding learning is to learn to predict context. For example, given the training sentence when word "stem" is present, the output node corresponding to "cell" should has the largest output value.

Feature Ranking and Aggregation

ANOVA: Analysis of variance (ANOVA) includes an F-*test* statistics measure for feature selection using variance based correlation analysis. As show in Eq. (4), The F-*test* is defined as the variance between treatments (*i.e.* partitioning of samples using some criteria) *vs.* variance across all samples. A feature with a higher ratio value indicates that the feature (or the group of features) contribute a larger dispersion in the data, implying that the feature is more important with respect the target label.

$$F = \frac{variance \;\; between \;\; Treatments}{variance \;\; within \;\; Treatments} \tag{4}$$

In order to use ANOVA for feature selection, given a dataset with N instances, denote N_l as the number of instances with class label $y = c_l$. For each feature f_j, we use $\bar{f}_j^l$ to denote its mean feature value of all instances labeled as c_l, and $\bar{f}_j^l$ denote mean feature values of all instances, which are defined as

$$\bar{f}_j = \frac{\sum_{l=1}^{L} N_l \times \bar{f}_j^l}{N}; \quad \bar{f}_j^l = \frac{\sum_{i,y_i=c_l} x_{i,j}}{N_l} \tag{5}$$

After that, for each feature f_j its F-*test* score in Eq. (4) is defined as

$$F_j = \frac{\sum_{l=1}^{L} N_l \times (\bar{f}_j^l - \bar{f}_j)^2/(L-1)}{\sum_{l=1}^{L} \sum_{i,y_i=c_l} (x_{i,j} - \bar{f}_j)^2/(N-1)} \tag{6}$$

The larger the F-*test* value in Eq. (6), the stronger the feature f_i is correlated to the target (class label).

ReliefF: ReliefF is a similarity based feature ranking approach, which estimates the quality of features according to how well their values distinguish between instances that are near to each other[5]. An instance, x_i is randomly selected and k of it's nearest neighbors are selected from the same class, these are nearest hits, H_j. k nearest neighbors of the opposite class are also selected, these are nearest misses, M_j. For each feature, f, the quality estimation will be decreased if the feature is differing between x_i and H_j. The quality estimate of f will be increased if the feature is differing between x_i and M_j[5]. The quality estimate is a measure of how well the feature separates the classes from each other. The quality estimate of each feature, f is defined as $W[f]$ and is calculated for each instance x_i using Eq. (7)[5]. Where k indicates the number of nearest neighbors to search for. For our implementation, $k = 10$. The contribution for each class of misses is weighted with prior probability of the class, $P(C)$ divided by a factor of $1 - P(class(x_i))$ which represents the sum of probabilities for the misses' class[5].

$$W[f] = W[f] - \sum_{j=1}^{k} \frac{diff(f, x_i, H_j)}{(m \times k)} + \sum_{C \neq class(x_i)} \left(\frac{\left[\frac{P(C)}{1 - P(class(x_i))} \sum_{j=1}^{k} diff(f, x_i, M_j) \right]}{(m \times k)} \right) \tag{7}$$

The function $diff(f, x_i, x_j)$ calculates the difference between the values of feature f for two instances x_i and x_j. For nominal attributes the function is defined in Eq. (8). For continuous attributes the function is defined in Eq. (9)[5].

$$diff(f, x_i, x_j) = \begin{cases} 0, & \text{if } value(f, x_i) = value(f, x_j) \\ 1, & \text{otherwise} \end{cases} \tag{8}$$

$$diff(f, x_i, x_j) = \frac{|value(f, x_i) - value(f, x_j)|}{max(f) - min(f)} \tag{9}$$

Mutual Information: Mutual Information (MI) measures the amount of information one random variable has about another variable[6]. MI will be equal to zero if two random variables are independent, higher values indicate higher-dependency. The concept of mutual information is tied to the concept of entropy. The entropy of a random variable y is denoted by $H(y)$, this measures the uncertainty of y and represents the total information conveyed by y. The MI between variables x and y is denoted as $I(x;y)$ and is defined in Eq. (10). This defines the information delivered from x to y is equal to the reduction of uncertainty of y when x is known[7].

$$I(X;Y) = \sum_{x \in X} \sum_{y \in Y} p(x,y) \frac{p(x,y)}{p(x)p(y)} \tag{10}$$

To estimate the MI for feature ranking, the Sklearn package implementation was used[2]. This implementation uses a nearest neighbor method to estimate MI between two variables. The method estimates $H(X)$ from the average distance to the k-nearest neighbor, averaged over all x_i. The estimate for MI is defined as follows[8].

$$I(X,Y) = \psi(k) - \frac{1}{k} - \langle \psi(n_x) + \psi(n_y) \rangle + \psi(N) \tag{11}$$

Where $\psi(x)$ is the digamma function. n_x and n_y denotes the number of x and y points in the region around each point i that contain k-nearest neighbors. N denotes the total number of points. And $\langle \ldots \rangle$ denotes the averages of all points $i \in [1, \ldots, N]$[8].

CIFE: Conditional Informative Feature Extraction (CIFE) maximizes information conveyed by the features by reducing the class-relevant redundancies.

The CIFE score for a specific feature, f_i, is denoted as $J_{CIFE}(f_i)$ and defined in Eq. (12)[7]. Where $I(f_i;c)$ represents the class relevant information of feature f_i and $R_c(f_j;f_i)$ represents the class-relevant redundancy between two features f_j and f_i.

$$J_{CIFE}(f_i) = \text{argmax} \left\{ \sum_{i=1}^{m} \left[I(f_i;c) - \sum_{j=1}^{m-1} R_c(f_j;f_i) \right] \right\} \tag{12}$$

This method provides an approximation of joint information between features with second-order interactions taken into account[7].

ICAP: ICAP (Interaction Capping)[9] is a mutual information based feature selection method, but it extends mutual information to evaluate interaction between than two variables to find feature set with minimized redundant interaction.

In order to quantify multi-information interaction between three variables, ICAP extends MI measure in Eq. (10) to reduce redundant interaction as shown in Eq. (13).

$$I(X;Y;Z) = \begin{cases} I(\{X,Y\};Z) - I(X;Z) - I(Y;Z) \\ I(Y;Z|X) - I(Y;Z) \end{cases} \tag{13}$$

By using Eq. (13), ICAP calculates the multi-information for each feature f_i using Eq. (14). A feature f_i's mutual information to the class label Y is penalized, if interaction between f_i, class label Y, and any subset of already selected features $s_j \in S$ is redundant (*i.e.*, $I(f_i;s_j;Y) < 0$).

$$J_{ICAP}(f_i) = I(f_i;Y) + \min_{s_i \in S}(0, I(f_i;s_j;Y)) \tag{14}$$

Dowdall Aggregation: Formally, given a dataset $\mathscr{D}$ with n instances $\mathscr{D} = \{x_1, x_2, \cdots, x_n\}$, each instance has m features $f_1, f_2, \cdots, f_m$. For each instance x_i, we use $x_{i,j}$ to denote the jth feature values of instance x_i, and y_i denotes the class label of

x_i. A filter approach ranks all features into an ordered list of features $\pi_i = [f_{-}^{1}, f_{-}^{2}, \cdots, f_{-}^{m}]$, where the superscript denotes the position in the ranked list of a certain feature f_{-}. For example f_{10}^{1} denotes that the original feature f_{10} is now ranked at the 1st place in the ranked feature list. In order to differentiate different filter approaches, we use π_i and π_j to denote different ranking order from filter method i and filter method j, respectively.

Dowdall system is assigns a fraction number, inverse to the ranking order, as the weight value for each ranking method. For each feature f_i, its Dowdall value is defined as

$$DA(f_i) = \sum_{j=1}^{n} \frac{1}{\pi_j(f_i)} \tag{15}$$

Dowdall method favors candidates with many first preferences (top ranking candidates). If a feature f_i is accidentally ranked to the bottom of the feature list by a method, it will have very little impact to f_i's DA aggregation value because it contributes a small fraction weight values to the final aggregation.

Feature Ranking Results: The results of aggregated feature ranking (using Dowdall Aggregation) are reported in Table 1, where a superscript (^{s},^{k},^{e}) denote a statistics feature, a keyword feature, and an embedding feature, respectively. The value in the parenthesis denotes Dowdall ranking. For example, "Eligibility Words^{s} (2)" denotes that this is a statistics feature, ranked no. 2 out of all 660 features. Embedding features belong to a vector of size 100 from the vector representation of the detailed description field. The feature names for embedding features represent their index position in the vector, {0:99}. The top ranked feature, "8^{e} (1)" is the 9th index position of the detailed description document vector. The left most column of Table 1 shows all 40 statistics features and their respective ranking. The middle column shows the top 40 keyword features and their respective ranking. The right column shows the top 40 ranked features out of all features.

Recursive Feature Elimination

Recursive feature elimination is used to demonstrate the performance of using all features for classificationIn recursive feature elimination, a classifier is trained m times, where m is the number of features in the full dataset. Using all features, $m = 640$. Using 5-fold cross validation, the classifier is tested and trained 5 times. After the test performance is determined for the feature set, the least significant feature is removed from m resulting in a new feature set m'. This is repeated until m' consists of a single feature. The scores from each iteration can then determine the optimal number of features to use. This process is repeated for each training set of the 5 datasets in the outer 5-fold cross validation split. The scores from each outer 5-fold cross validation are then averaged together to determine the averaged AUC scores using increasing number of features. Recursive feature elimination utilized a random forest classifier with the same optimized parameters as used in termination classification models; 1000 fully grown trees, Gini criterion, $\sqrt{m}$ features for best split, $n = 2$ samples to split an internal node, $n = 4$ samples required for a leaf node and samples were not Bootstrapped.

Figure 2 displays the averaged nested 5-fold cross validation scores for recursive feature elimination. The results display a maximum AUC score of 71.22% with 538 features selected. Using all features, the AUC score was 71.15%. Since the lowest 102 ranked features do not significantly decrease AUC scores, these results indicate that utilizing all features does provide the highest performance. This validates using all feature combinations has the greatest predictive power for clinical trial termination.

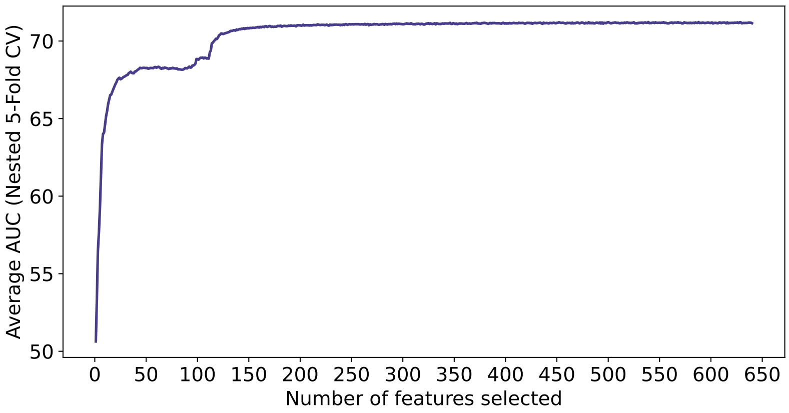

Figure 2. Recursive Feature Elimination results. The X-axis denotes the number of features selected at each step. The Y-axis denotes averaged AUC scores over nested 5-fold Cross Validation.

Statistics Features	Keyword Features	All Features
Eligibility Words^s (2)	Verrucous^k (6)	8^e (1)
No Eligibility Requirement^s (3)	Testicular^k (8)	Eligibility Words^s (2)
Inclusion Words^s (5)	Neuroblastoma^k (13)	No Eligibility Requirement^s (3)
Number Countries^s (7)	Sezary^k (20)	1^e (4)
Phase 1^s (10)	Fungoides^k (27)	Inclusion Words^s (5)
Eligibility Lines^s (11)	Nasopharynx^k (31)	Verrucous^k (6)
Number Arms^s (12)	Mycosis^k (32)	Number Countries^s (7)
Industry Sponsor^s (14)	Contiguous^k (33)	Testicular^k (8)
Average Inclusion Words^s (15)	Germ^k (39)	13^e (9)
Average Eligibility Words^s (17)	Thyroid^k (42)	Phase 1^s (10)
Exclusion Words^s (18)	Noncontiguous^k (48)	Eligibility Lines^s (11)
Number Officials^s (19)	Paranasal^k (50)	Number Arms^s (12)
Average Exclusion Words^s (21)	Myelomonocytic^k (51)	Neuroblastoma^k (13)
Random Groups^s (22)	Hypopharynx^k (57)	Industry Sponsor^s (14)
Eligibility Numbers^s (24)	Uterine^k (60)	Average Inclusion Words^s (15)
Inclusion Lines^s (25)	NSCLC^k (61)	16^e (16)
Exclusion Lines^s (26)	Oropharynx^k (63)	Average Eligibility Words^s (17)
Healthy Volunteer^s (28)	AML^k (71)	Exclusion Words^s (18)
Exclusion numbers^s (34)	Salivary^k (73)	Number Officials^s (19)
Responsible Party: Sponsor^s (37)	Remission^k (74)	Sezary^k (20)
Inclusion Numbers^s (41)	Cancer^k (77)	Average Exclusion Words^s (21)
No Phase^s (43)	Valve^k (78)	Random Groups^s (22)
Number Sites^s (44)	Esophagus^k (80)	18^e (23)
Phase 2^s (45)	Sepsis^k (82)	Eligibility Numbers^s (24)
Number Collaborators^s (53)	Larynx^k (86)	Inclusion Lines^s (25)
Main Country: USA^s (54)	Neck^k (88)	Exclusion Lines^s (26)
Has Expanded Access^s (56)	Epilepsy^k (90)	Fungoides^k (27)
Has DMC^s (70)	Endometrial^k (91)	Healthy Volunteer^s (28)
Has Oversight^s (72)	Multiple^k (94)	22^e (29)
Uses Blinding^s (83)	Cleaved^k (95)	24^e (30)
Responsible Party: Investigator^s (89)	Relapsed^k (96)	Nasopharynx^k (31)
Placebo Group^s (98)	Astrocytoma^k (99)	Mycosis^k (32)
Industry Collaborator^s (102)	Esophageal^k (101)	Contiguous^k (33)
Responsible Party: None Listed^s (106)	Extranodal^k (103)	Exclusion numbers^s (34)
Phase 3^s (108)	Efficacy^k (105)	25^e (35)
Interventional Study^s (114)	Degeneration^k (107)	28^e (36)
FDA Regulation^s (117)	Pressure^k (109)	Responsible Party: Sponsor^s (37)
Gender Restriction^s (126)	Infusion^k (110)	31^e (38)
Phase 4^s (129)	Marrow^k (112)	Germ^k (39)
Age Restriction^s (153)	Gland^k (115)	81^e (40)

Table 1. Features and their aggregated ranking using Dowdall Aggregation. The superscripts (^{s},^{k},^{e}) denote feature types (statistics features, keyword features, or word embedding features, respectively). The number in the parenthesis denotes the aggregated ranking of the feature, with (1) being the best ranking. The top 40 of Statistics Features, Keyword Features and All Features are shown.

Classification Methods

Neural Network: We use multi-layer feed forward neural network in our experiments. The network consists of three layers, an input layer, a hidden layer with 100 nodes, and an output layer with a single node (which classifies each clinical trial as "terminated" or "completed"). Each node i, connected to node, j has associated weight w_{ij} and associated bias input b_i[10]. In the forward pass, each node in the hidden layer and output layer compute a weighted sum of inputs, a_i, and apply the sigmoid activation function to a_i to produce the output y_i, as defined in Eqs. (16) and (17)[11].

$$a_i = \sum_{j=1}^{n} (w_j \cdot x_j) + b_i \tag{16}$$

$$y_i = \frac{1}{1+e^{-a_i}} \tag{17}$$

In the second phase of training, the neural network utilized the *Adam* optimization function[12] to minimize the loss function and update weights. The loss function, $L(\theta_w)$, is defined in Eq. (18)[13].

$$L(\theta_w) = -\frac{1}{N}\sum_{i=1}^{N}\Big[y_i \times \log\big(h_\theta(x_i)\big) + (1-y_i) \times \log\big(1-h_\theta(x_i)\big)\Big] \tag{18}$$

Neural Network parameters are optimized first with a randomized grid search, then an exhaustive grid search.The randomized grid search is used to determine the optimal values for the hidden layer activation function, batch size, training epochs and number of nodes in the hidden layer. To perform randomized grid search, for each training dataset from 5-fold cross validation, 30 different combinations of varying values for the parameters were tested using nested cross validation. Each outer fold returned a parameter combination that is determined as optimal. Due to the random nature of randomized grid search, not all parameter combinations are tested, thus in some cases, multiple parameter values are suggested as optimal. These values are listed in Table 2 (a). To determine the final optimal parameter values for Neural network, an exhaustive grid search tested the two different batch size and node parameter values. The exhaustive grid search results are shown in Table 2 (b). From the exhaustive grid search, batch size of 80 and 100 nodes are selected as the optimal parameters, due to their superiority in AUC values.

Parameter	Value
Hidden Layer Activation Function	Sigmoid
Batch Size	40, 80
Epochs	10
Nodes	50, 100

(a) Optimal Parameters from Randomized Grid Search

Batch Size	Nodes	Accuracy	Balanced	F1	AUC
80	100	88.48%	50.16%	0.89%	71.38%
80	50	88.51%	50.10%	0.54%	71.35%
40	50	88.47%	50.19%	1.01%	71.18%
40	100	88.41%	50.24%	1.42%	70.91%

(b) Exhaustive grid search results

Table 2. Neural Network Grid Search. (a) A randomized grid search suggested the parameter values listed as optimal; (b) testing the suggested parameter values, an exhausted grid search determined the final optimal values for batch size and nodes. The average nested 5-fold cross validation scores are shown.

Random Forest: Random forests are ensembles of decision tree classifiers. A random forest classifier consists of K trees; $\{h(x,\Theta_k), k = 1,\ldots,K\}$. Each tree is generated from independent random vectors, $\{\Theta_k\}$, from samples in the training set[14]. The tree classifiers are then combined by averaging their probabilistic predictions[2].

Random forests use κ additive functions to predict output, $\hat{y}$, as defined in Eq. (19). Where $\mathscr{F} = \{f(x) = w_q(x)\}$; q represents the structure of each tree in the random forest. f_k corresponds to an independent tree structure q with weights w[15].

$$\hat{y}_i = \phi(x_i) = \sum_{k=1}^{\kappa} f_k(x_i); \quad f_k \in \mathscr{F} \tag{19}$$

Preliminary tests with increasing sizes of K trees, K = 100, K = 500 and K = 1000 indicate that increased K provided the highest performance. To further optimize parameters for Random Forest, a randomized grid search tests 30 different combinations of values for the number of trees, the minimum number of samples required to split an internal node, the minimum samples required to be at a leaf node and to use bootstrap samples to build trees. The parameter values listed in Table 3 (a) are those that randomized grid search returned as optimal. To determine the final optimal parameters for Random Forest, an exhaustive grid search tested 1000, 1500, and 2000 trees. The nested 5-fold cross validation scores from exhaustive search are shown in Table 3 (b). This determined $K = 1000$ is the optimal number of trees.

The final random forest implementation in our study uses 1,000 trees group to full extent. The Gini criterion was used to determine the best split. The number of features considered for best split was set to $\sqrt{m}$, where m is the number of features in the training dataset. A minimum of 2 samples are required to split an internal node. A minimum of 4 samples are required to be at a leaf node. The random forests do not bootstrap samples to build the trees, the whole training dataset is used to build each tree.

Parameter	Value
Number of Trees	1000, 1500, 2000
Minimum samples required to split an internal node	2
Minimum samples required to be at a leaf node	4
Bootstrap Samples	False

(a) Optimal Parameters from Randomized Grid Search

# Trees	Accuracy	Balanced	F1-Score	AUC
1000	88.48%	50.07%	0.28%	71.56%
1500	88.48%	50.07%	0.28%	71.54%
2000	88.48%	50.07%	0.28%	71.53%

(b) Exhaustive grid search results

Table 3. Random Forest Grid Search. (a) A randomized grid search suggested the parameter values listed as optimal; (b) testing the suggested parameter values, an exhausted grid search determined the final optimal values for number of trees. The average nested 5-fold cross validation scores are shown.

XGBoost: XGBoost stands for Extreme Gradient Boosting. It is a decision tree ensemble algorithm that uses a gradient boosting framework. XGBoost minimizes the objective function defined in Eq. (20), where $\hat{y}_i^{(t)}$ is the prediction of the i-th instance at iteration t[15], and $f_t(x_i)$ denotes the prediction of a classification tree f_t on instance x_i. l is a differentiable convex loss function that measures difference between predicted label, $\hat{y}_i$ and target label, y_i. In gradient tree boosting, f_t is greedily added to improve the model[15].

$$\mathscr{L}^{(t)} = \sum_{i=1}^{n} l\left(y_i, \hat{y}_i^{(t-1)} + f_t(x_i)\right) + \Omega(f_t) \tag{20}$$

The regularization term, $\Omega(f)$, defined in Eq.(21), penalizes the complexity of tree f_t with T leaves. Nodes in tree f_t are split if there is a positive reduction in the loss function, γ is the minimum loss reduction to continue splits of leaf nodes. Increasing values of γ increases the complexity costs with each additional leaf.

$$\Omega(f) = \gamma T + \frac{1}{2}\lambda \parallel w \parallel^2 \tag{21}$$

To optimize parameters for XGB, a randomized grid search tested 30 different combinations of values for the number of trees, γ, the subsample ratio, feature sampling, maximum depth of trees and minimum weight in child nodes. When building trees, the subsample ratio determines the ratio of of training instances used to build each tree. The feature sampling determines the fraction of features that are randomly selected to train each tree. The suggested optimal parameters from randomized grid search is listed in Table 4. To determine the final optimal parameters, these are used in an exhaustive grid search. Table 5 lists the results from average nested 5-fold cross validation in grid search. The final parameters with XGBoost in our report are 300 trees, $\gamma = 5$, maximum depth of 4, minimum weight of 5, subsample ratio of 1 (all training samples used), and feature sampling ratio of 0.6. These features were choosen due to their superiority in AUC scores.

Parameter	Value
Number of Trees	100, 300
Minimum loss reduction (γ)	2, 5
Subsample Ratio	1
Feature Sampling	0.6
Maximum Depth	4, 5
Minimum weight in a child node	1, 5

Table 4. Optimal Parameters from Randomized Grid Search for XGBoost.

# Trees	γ	Max. Depth	Min. Weight	Accuracy	Balanced	F1-score	AUC
300	5	4	5	88.52%	50.25%	1.20%	72.53%
300	2	4	5	88.51%	50.25%	1.23%	72.49%
300	5	5	1	88.51%	50.35%	1.68%	72.45%
300	2	4	1	88.52%	50.27%	1.31%	72.44%
300	5	4	1	88.52%	50.24%	1.15%	72.41%
300	2	5	5	88.51%	50.35%	1.68%	72.41%
100	5	5	1	88.54%	50.13%	0.59%	72.40%
100	2	5	1	88.54%	50.12%	0.58%	72.39%
300	5	5	5	88.51%	50.34%	1.60%	72.39%
100	5	5	5	88.55%	50.11%	0.49%	72.38%
100	2	5	5	88.54%	50.10%	0.45%	72.37%
300	2	5	1	88.50%	50.38%	1.79%	72.34%
100	5	4	5	88.55%	50.05%	0.21%	72.17%
100	2	4	5	88.54%	50.04%	0.20%	72.15%
100	2	4	1	88.54%	50.04%	0.19%	72.13%
100	5	4	1	88.54%	50.03%	0.15%	72.07%

Table 5. XGBoost exhaustive grid search results to determine the number of trees, γ, maximum depth of the trees and minimum weight in child nodes. The averaged nested 5-fold cross validation scores are shown.

Logistic Regression: Logistic Regression is a nonlinear classification model. The probabilities describing the possible classification of a single trial are modeled using a logistic function, as defined in Eq. (22), where x is the training data, y is the class label, and w is the weight vector[16].

$$\mathscr{P}w(y = \pm 1|x) \equiv \frac{1}{1+e^{-yw^T x}} \tag{22}$$

A binary class l_2 penalized logistic regression minimizes the following cost function in Eq. (23), where $C > 0$ is a penalty parameter[16].

$$\mathscr{P}(w) = C\sum_{i=1}^{n} \log\left(1+e^{-yw^T x_i}\right) + \frac{1}{2}w^T w \tag{23}$$

Classification Framework

Random Under Sampling: After features are created and optimal parameters are determined, different rates of random under sampling are tested to determine the optimal ratio of random under sampling. Note that feature normalization was also tested, however normalization did not improve classification performance.

Figure 4 displays averaged Accuracy, Balanced Accuracy, F1 and AUC scores from 5-fold cross validation using different rates of random under sampling. The original dataset has an imbalanced ratio of 7.75:1, thus the first point displays no random undersampling, (*i.e.* single models). Sampling rates of 7:1, 6:1, 5:1, 4:1, 3:1, 2:1, 1:1, and 0.9:1 are considered. The sampling rate measures the ratio of completed trials to terminated trials. As the imbalanced ratio decreases, Balanced Accuracy, F1-score and AUC all show increases. At a rate of 0.9:1 (terminated trials outnumber completed trials), F1-score and Balanced Accuracy begin to decrease. For XGBoost and Neural network, there is a minor decrease (<0.5%) in AUC seen in 1:1 sampling rate. Due to the superiority of Balanced Accuracy and F1-score, 1:1 sampling rate was chosen to report in our final ensemble results in the manuscript. Accuracy score decreases at 2:1 sampling, due to the models increasingly classifying clinical trials as Terminated, which will incorrectly classify some completed trials.

Ensemble Learning: The classification framework is shown in Algorithm (1). After the features are created, for each combination of features, random under sampling is performed 10 times. For our final ensemble method, the majority class is under sampled at a rate of 1 times the minority class. This represents an even ratio of completed and terminated trials. Each classifier from random undersampling is combined by averaging the classifiers output probabilities from the test dataset. The averaged probabilities constitute the final prediction of the test data set. The entire dataset is split into 5 validation sets and the frame work was completed for each set. The resulting performance scores are averaged to determine the final performance metrics for each model.

Algorithm 1 Clinical Trial Analtyics and Prediction Framework

input: (1) Clinical trial reports from ClinicalTrials.gov; (2) Random under sampling (RUS) times: κ; (3) Embedding feature dimensions: d

output: $\hbar(\cdot)$: Clinical trial termination predictive model.

$\mathscr{D} \leftarrow$ Apply inclusion criteria from Figure 3.

$\{\mathscr{D}^{+}, \mathscr{D}^{-}\} \leftarrow$ Label positive (+) and negative (-) trials in $\mathscr{D}$

$\mathbb{F}_s^{\mathscr{D}} \leftarrow$ Create statistics features from $\mathscr{D}$

$\mathbb{F}_k^{\mathscr{D}} \leftarrow$ Create keyword features from $\mathscr{D}$

$\mathbb{F}_e^{\mathscr{D}} \leftarrow$ Create embedding features from $\mathscr{D}$

$\mathbb{F}^{\mathscr{D}} \leftarrow \{\mathbb{F}_s^{\mathscr{D}} \oplus \mathbb{F}_k^{\mathscr{D}} \oplus \mathbb{F}_e^{\mathscr{D}}\}$ Concatenate all features

$\hbar(\cdot) \leftarrow \emptyset$

for each round of random under sampling (RUS) $k \in \kappa$ **do**

  $\hat{\mathscr{D}}^{-} \leftarrow$ Random under sampling on $\mathscr{D}^{-}$

  $\hat{\mathscr{D}} \leftarrow \{\mathscr{D}^{+} \cup \hat{\mathscr{D}}^{-}\}$. Create balanced training set

  $\hbar_k(\cdot) \leftarrow$ Train classifier from $\hat{\mathscr{D}}$ using features $\mathbb{F}^{\mathscr{D}}$

  $\hbar(\cdot) \leftarrow \hbar(\cdot) \cup \hbar_k(\cdot)$

end for

return $\hbar(\cdot)$.

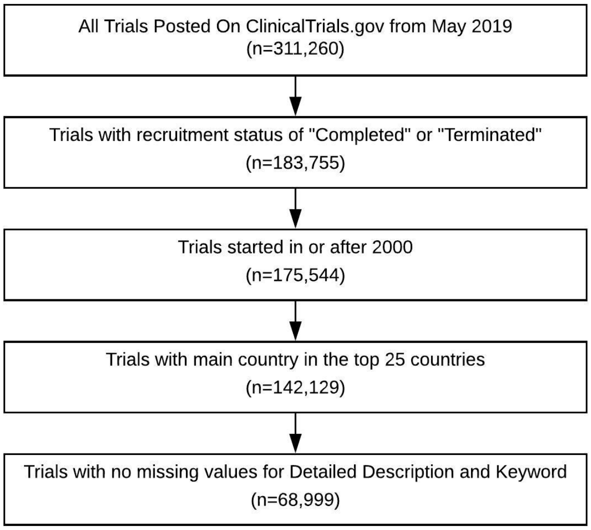

Figure 3. Clinical trial inclusion criteria

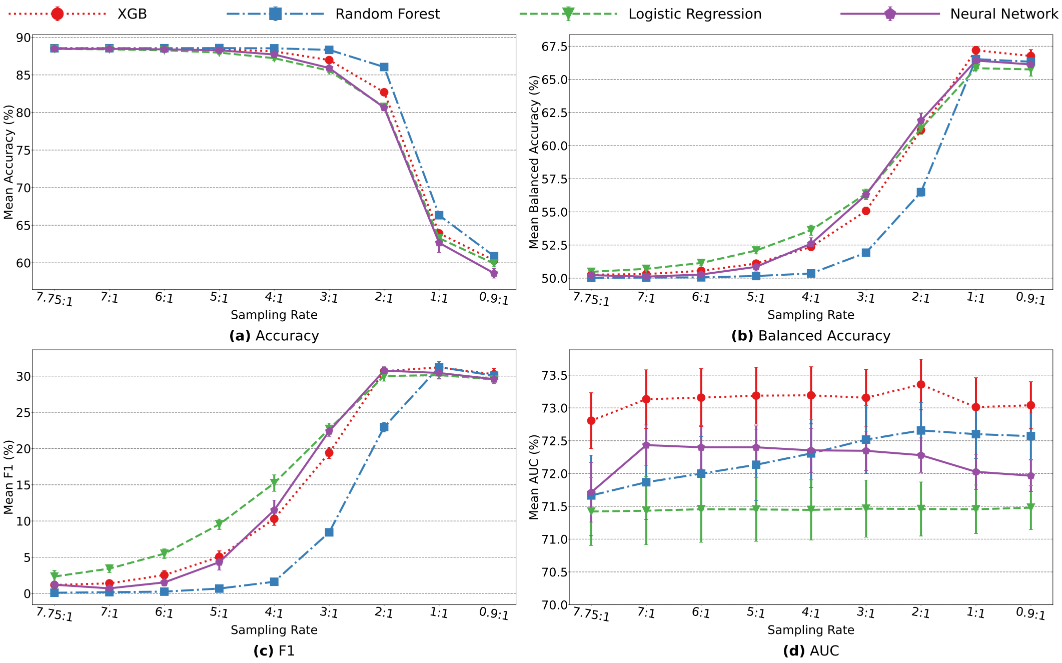

Figure 4. Mean accuracy (a), Balanced Accuracy (b), F1-score (c), and AUC-score (d) for random under sampling the data set at different completed to terminated trials ratios after 5-fold cross validation. The x-axis indicates the sampling rate, and y-axis represents the performance measures. 7.75:1 indicates no random under sampling (original sample ratios between Completed *vs.* Terminated trials, and 1:1 indicates an even sampled ratio.

Performance Metrics

Confusion Matrix: In our study, terminated clinical trials are listed as "Positive" Class, as they are the class we wish to predict for. If a clinical trial is terminated and the model predicts it as terminated, it is considered a True Positive (TP). If a clinical trial is terminated and the model predicts it as completed, this represents a False Negative (FN). Similarly, if a trial is completed and the model predicts it as completed, this represents a True Negative (TN). If the trial is completed and the model predicts it as terminated, this represents a False Positive (FP). A confusion matrix representing these concepts is shown in Figure 5.

		Predicted	
		Completed	Terminated
Actual	Completed	True Negatives (TN)	False Positives (FP)
	Terminated	False Negatives (FN)	True Positives (TP)

Figure 5. A graphical depiction of a confusion matrix, a table that describes the performance of a classifier

Classification Accuracy: Accuracy is the ratio of all true positives and true negatives to all classes. With class imbalanced data sets, accuracy can be high without making useful predictions. The model could have accuracy as high as 88.54% while not even predicting one clinical trial as terminated. F1-score and AUC provide more insight on the performance of a model with imbalanced datasets.

$$\text{Accuracy} = \frac{\text{TP}+\text{TN}}{\text{TP+TN+FP+FN}} \tag{24}$$

F1-Score: F1-score, Eq. (25) is a weighted average of Precision and Recall. Precision is the ratio of correctly predicted positive classes to total predicted positive classes, and recall is the ratio of correctly positive predicted classes to all positive classes[17].

$$\text{F1-Score} = \frac{2\times(\text{Recall}\times\text{Precision})}{(\text{Recall}+\text{Precision})}; \qquad \text{Precision} = \frac{\text{TP}}{\text{TP+FP}}; \qquad \text{Recall} = \frac{\text{TP}}{\text{TP+FN}} \tag{25}$$

Balanced Accuracy: Balanced Accuracy, Eq.(26) is the average of True Negative Rate (TNR) and True Positive Rate (TPR). TPR is also known as Recall. This metric can be useful in the cases of class imbalance.

$$\text{Balanced Accuracy} = \frac{\text{TNR}+\text{TPR}}{2}; \qquad \text{TNR} = \frac{\text{TN}}{\text{TN+FP}}; \qquad \text{TPR} = \frac{\text{TP}}{\text{TP+FN}} \tag{26}$$

AUC Values: The classifiers output is represented as a probability that a clinical trial will be terminated or completed. A specific threshold is stated (0.5) where if the clinical trial is at the threshold or above, it is classified as terminated. If the clinical trial is below the threshold, it is classified as completed. A Receiver Operating characteristic Curve (ROC curve) is a graph that displays the performance of a binary classifier as the threshold changes. The ROC curve will show how the number of correctly classified terminated trials will vary with the number of incorrectly classified completed trials. The ROC curve plots the False Positive Rate (FPR) on the x-axis and the True Positive Rate (TPR) on the y-axis. FPR and TRP are defined in Eq. (27) [17].

$$\text{FPR} = \frac{\text{FP}}{\text{FP+TN}}; \qquad \text{TPR} = \frac{\text{TP}}{\text{TP+FN}} \tag{27}$$

AUC is the area under the ROC curve and relates to the ranking quality of the classification. For a given classifier, the AUC can be formally defined as A in Eq. (28)[18]. Where $x_1, x_2 \ldots, x_{pos}$ is the output of the classifier on the positive (terminated) examples; and $y_1, y_2, \ldots, y_{neg}$ is the output of the classifier on the negative (completed) examples.

$$A = \frac{\sum_{i=1}^{pos}\sum_{j=1}^{neg} 1_{x_i > y_j}}{pos \times neg} \tag{28}$$

The AUC, A, is the value of the Wilcoxon-Mann-Whitney statistic. This can be views as a measure based on pairwise comparisons between classifications of the two classes[18]. If the threshold is perfect then all terminated trials will be ranked higher than completed trials and AUC will equal 1. Deviations from this ranking will decrease the AUC. An AUC value of 0.5 implies random ranking. AUC has been shown to be statistically consistent and more discriminating measurement compared to accuracy[19]. AUC has also been an accepted measurement for datasets with class-imbalances[20].

Confusion Matrices

To demonstrate the performance of single models *vs.* ensemble models, the classification confusion matrices are displayed in Figure 6 and Figure 7, respectively. Confusion matrices are obtained after averaged 5-fold cross validation results.

The single model methods only obtain 1-18 true positives. With the low true positive rate, there is also a high true negative rate, as models only incorrectly classify 1-31 completed trials. The harmony between these two result in 50% balanced accuracy scores seen with single model methods.

Ensemble models show dramatic increases in true negative samples, from 1056-1120. This causes the large increase in F1-scores and Balanced Accuracy scores seen with Ensemble models. While the true negative rate has increased, there's a concurrent increase in False Positives. As the models increasingly classify clinical trials as terminated, they begin to incorrectly classify more completed trials. This is why there is a decrease in overall accuracy scores of ensemble models.

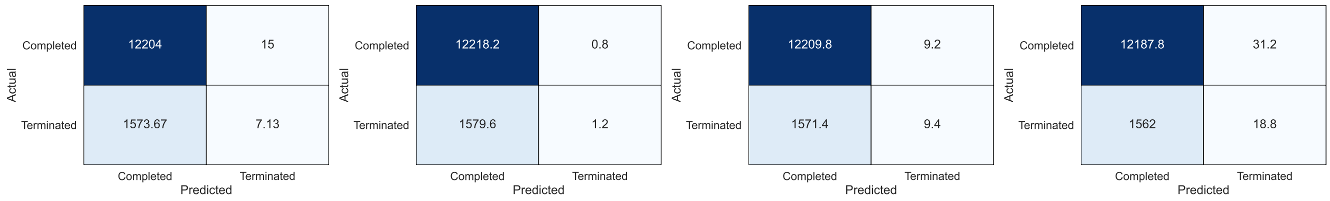

(a) Neural Network **(b)** Random Forest **(c)** XGBoost **(d)** Logistic Regression

Figure 6. Confusion matrices after averaged 5-fold cross validation for Single Models

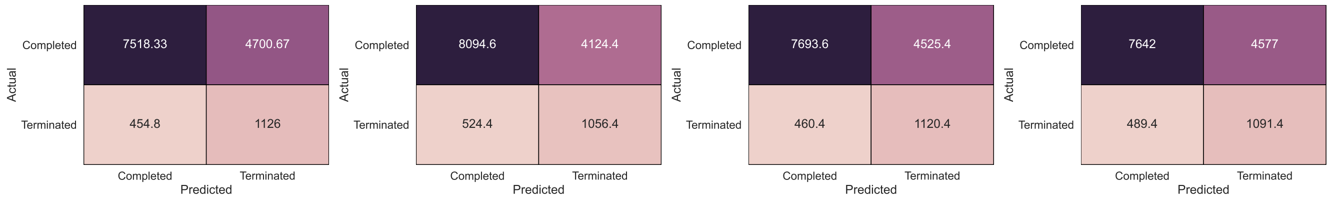

(a) Neural Network **(b)** Random Forest **(c)** XGBoost **(d)** Logistic Regression

Figure 7. Confusion matrices after averaged 5-fold cross validation for Ensemble Models

Statistical Tests

Friedman test: A Friedman test is a non-parametric statistical test that can be used to compare the results of multiple algorithms across different data sets[21]. The tests considers the average rank of each algorithm on the compared datasets. Classifiers are ranked in descending order, thus the classifier with highest scores for a dataset will be ranked as 1. In the case of a tie between two scores, the average rank is assigned, (*i.e.* if two classifiers tie for 2nd place, the assigned ranks are 2.5). Under the null hypothesis, there is no difference between algorithms, thus their average ranks will not be different. Considering k classifiers tested on n datasets, the average rank of the j-th classifier is R_j as defined in Eq.(29), where r_i^j is the rank for classifier j on dataset i. In our analysis, we have 4 classifiers and 7 datasets (from the different combinations of features). The Friedman statistic is defined by χ_F^2, in Eq.(30).

$$R_j = \frac{1}{n}\sum_{i=1}^{n} r_i^j \tag{29}$$

$$\chi_F^2 = \frac{12n}{k(k+1)}\left[\sum_{j=1}^{k} R_j^2 - \frac{k(k+1)^2}{4}\right] \tag{30}$$

After rejecting the null-hypothesis that the classifiers are equivalent, we perform the Nemenyi post-hoc test for pairwise comparisons of performance. Two classifiers are significantly different if the average ranks differ by the critical difference,

CD, defined in Eq.(31). Where q_α is the Studentized range statistic divided by $\sqrt{2}$[21]. In our study, with $k = 4$ classifiers and $\alpha = 0.05$, $q_{0.05} = 2.569$. With a lower level of confidence, $\alpha = 0.10$, $q_{0.10} = 2.291$. Since there are $n = 7$ datasets, with $\alpha = 0.05$, $CD = 1.773$; with $\alpha = 0.10$, $CD = 1.581$.

$$CD = q_\alpha \sqrt{\frac{k(k+1)}{6n}} \tag{31}$$

The results from the Nemenyi post-hoc test can be displayed in a critical difference diagram, such as seen in Figures 8,9,10 and 11. The top line demonstrates the average ranks, R_j of each classifier. Since classifiers are ranked in descending order, those listed on the left (closer to 1) have higher performance than those on the right. At the top of the diagram, the critical difference, CD, length is displayed. The critical difference diagrams demonstrate pairwise comparisons of all classifiers. Classifiers that are not significantly different, (*i.e.* their average ranks do not differ by CD), are grouped together with a bar.

The Friedman test results for Accuracy, Balanced Accuracy, F1-score and AUC scores with respect to single models and Ensemble models are shown in Table 6. For single models, a Friedman test determined that the models were only significantly different with respect to Accuracy scores. A Nemenyi post-hoc test, with $\alpha = 0.05$, Figure 8 (a) shows Random Forest, XGBoost and Logistic Regression grouped together. This indicates that these three models are not significantly different in accuracy; and Random Forest (which is closer to 1) is significantly better than Neural Network. Since XGBoost, Logistic Regression and Neural Network are also combined with a bar, they are not significantly different to each either with respect to accuracy. Using a lower confidence interval, $\alpha = 0.1$, Figure 8 (b), Random Forest is significantly better than Logistic Regression and Neural Network; XGBoost is significantly better than Neural Network. There is no significant difference between Random Forest and XGBoost; no significant difference between XGBoost and Logistic Regression; and no significant difference between Logistic Regression and Neural Network.

	Single Models		Ensemble Models	
	χ^2_F	p	χ^2_F	p
Accuracy	12.429	0.006	10.029	0.018
Balanced	1.80	0.615	7.971	0.047
F1	2.829	0.419	11.229	0.011
AUC	4.543	0.208	9.686	0.021

Table 6. Friedman test comparing the Accuracy, Balanced Accuracy, F1-Score and AUC with respect to single models and Ensemble models performance on the seven different combinations of features.

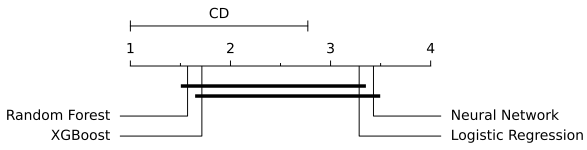

(a) $\alpha = 0.05$

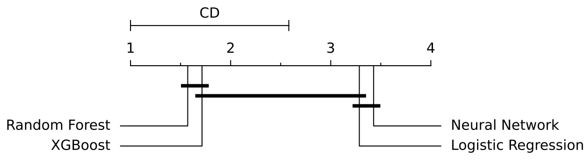

(b) $\alpha = 0.1$

Figure 8. Critical difference diagram for single model method comparing the four classifiers Accuracy scores on different combinations of features, (a) with $\alpha = 0.05$, $CD = 1.773$; (b) with $\alpha = 0.1$, $CD = 1.581$. Groups of classifiers that are not significantly different are connected.

For Ensemble models, the Friedman tests demonstrate that models are significantly different in all measures, as listed in Table 6. A Nemenyi post-hoc test, at $\alpha = 0.05$, for Accuracy in Figure 9 (a) demonstrates that Random Forest is statistically and significantly better than Neural Network. The other models are not significantly different. The Nemenyi post-hoc test, at $\alpha = 0.05$ for Balanced Accuracy in Figure 9 (a), demonstrates that Random Forest is statistically and significantly better than Logistic Regression. Note that for Balanced Accuracy and Accuracy post-hoc tests, decreasing the confidence interval does not change the results.

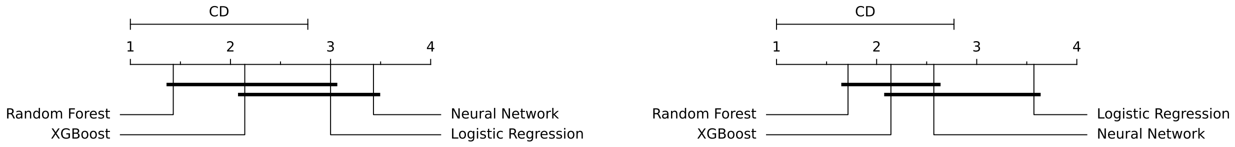

(a) Accuracy **(b)** Balanced Accuracy

Figure 9. Critical difference diagram for Ensemble model method comparing the four classifiers AUC scores on different combinations of features, (a) Accuracy; (b) Balanced Accuracy. Both diagrams display $\alpha = 0.05$, $CD = 1.773$. Groups of classifiers that are not significantly different are connected.

For Ensemble Models F1-scores, the Nemenyi post-hoc test determined that Random Forest is statistically and significantly better than Logistic Regression, Figure 10. When lowering the confidence interval to $p = 0.1$, Random Forest is significantly better than Neural network and Logistic Regression. There is no difference between XGBoost, Neural Network and Logistic Regression.

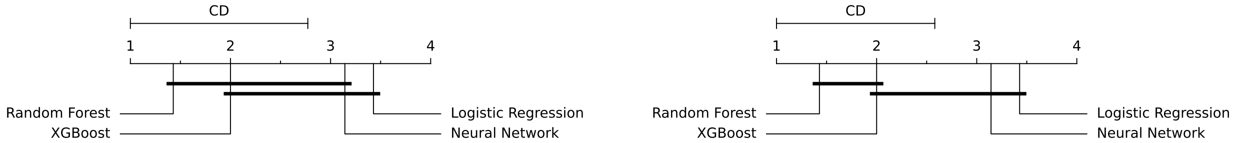

(a) $\alpha = 0.05$ **(b)** $\alpha = 0.1$

Figure 10. Critical difference diagram for Ensemble model method comparing the four classifiers F1-scores on different combinations of features, (a) with $\alpha = 0.05$, $CD = 1.773$; (b) with $\alpha = 0.1$, $CD = 1.581$. Groups of classifiers that are not significantly different are connected.

For Ensemble models AUC scores, the Nemenyi post-hoc test determined that Random Forest is statistically better and different than Logistic Regression, as shown in Figure 11. When lowering the confidence interval to $p = 0.1$, Random Forest and XGBoost are both statistically and significantly better than Logistic Regression. There is no statistical difference between Random Forest and XGBoost; there is no statistical difference between Neural Network and any of the models.

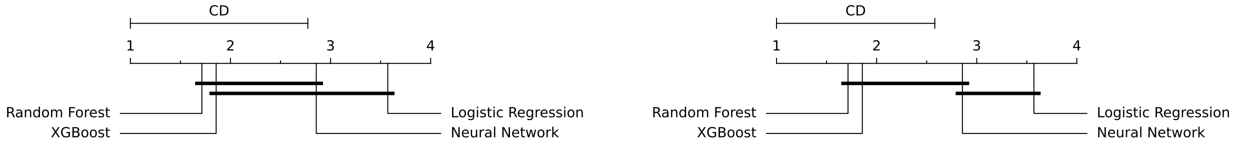

(a) $\alpha = 0.05$ **(b)** $\alpha = 0.1$

Figure 11. Critical difference diagram for Ensemble model method comparing the four classifiers AUC scores on different combinations of features, (a) with $\alpha = 0.05$, $CD = 1.773$; (b) with $\alpha = 0.1$, $CD = 1.581$. Groups of classifiers that are not significantly different are connected.

Corrected resampled t-test:The Friedman tests and Nemenyi post-hoc tests demonstrate the classifiers performance over the different combinations of features. These tests are not appropriate when doing a direct pairwise comparison of two classifiers on the same dataset, or the same classifier using two different feature combinations. For our study, to do pairwise comparisons, the corrected resampled t-test was used. This test is used in three places: (1) to determine significant differences between the single model classifiers and their ensemble model counterparts; (2) to demonstrate the statistical significance of using all features pairwise to each individual feature combination for respective models; (3) to verify the statistical significance of XGBoost ensemble models with all features compared to other ensemble models with all features.

The corrected resampled t-test was initially proposed to compare the performance of two classifiers. This test takes the variability of overlapping training and test sets into account[22] and was shown to have high replicability[23].

In k-fold cross validation, the whole dataset is split into k groups of training and test samples. The classifiers are run k times, while learning on training samples and testing performance on test samples. For each run, n_1 samples are used for training and n_2 samples are used for testing. The corrected resampled t-test is defined by t in Eq.(32)[23]. Where X_j is the difference between the performance scores of two algorithms on run j and $\hat{\sigma}^2$ is the variance of differences between performance scores for all runs.

$$t = \frac{\frac{1}{k}\sum_{j=1}^{k} x_j}{\sqrt{\left(\frac{1}{k} + \frac{n_2}{n_1}\right)\hat{\sigma}^2}} \tag{32}$$

XML schema and Statistics Feature Summary

A summary of all created statistics features is shown in Table 7. These fields are created directly from information from the XML fields for each clinical trial. A summary of all XML fields used for feature engineering and analysis is shown in Table 8. The keyword features are created directly from the keyword XML field, `keyword`. The embedding features are created from the detailed description text block from the detailed description field, `detailed_description/textblock`. `start_date` is used for our inclusion criteria, as described in Figure 3. `condition_browse/mesh_term` and `intervention_brows/mesh_term` are used in analysis of clinical trial research areas. All other XML fields were used to create statistic features.


```xml
- <clinical_study>
    <!-- This xml conforms to an XML Schema at: https://clinicaltrials.gov/ct2/html/images/info/public.xsd -->
  - <required_header>
      <download_date>ClinicalTrials.gov processed this data on July 17, 2019</download_date>
      <link_text>Link to the current ClinicalTrials.gov record.</link_text>
      <url>https://clinicaltrials.gov/show/NCT01650194</url>
    </required_header>
  - <id_info>
      <org_study_id>9785-CL-0011</org_study_id>
      <nct_id>NCT01650194</nct_id>
    </id_info>
    <brief_title>A Study to Determine Safety and Tolerability of Enzalutamide (MDV3100) in Combination With Abiraterone Acetate in Bone
      Metastatic Castration-Resistant Prostate Cancer Patients</brief_title>
    <official_title>A Phase 2 Study Determining Safety and Tolerability of Enzalutamide (Formerly MDV3100) in Combination With Abiraterone
      Acetate in Bone Metastatic Castration-Resistant Prostate Cancer Patients</official_title>
  - <sponsors>
    - <lead_sponsor>
        <agency>Astellas Pharma Global Development, Inc.</agency>
        <agency_class>Industry</agency_class>
      </lead_sponsor>
    - <collaborator>
        <agency>Medivation LLC, a wholly owned subsidiary of Pfizer Inc.</agency>
        <agency_class>Industry</agency_class>
      </collaborator>
    </sponsors>
    <source>Astellas Pharma Inc</source>
  - <oversight_info>
      <has_dmc>No</has_dmc>
      <is_fda_regulated_drug>Yes</is_fda_regulated_drug>
      <is_fda_regulated_device>No</is_fda_regulated_device>
    </oversight_info>
  - <brief_summary>
      <textblock> The purpose of this study was to explore the safety and tolerability of enzalutamide in combination with abiraterone acetate plus
        prednisone. Subjects diagnosed with cancer of the prostate that was getting worse and spreading to the bone despite receiving hormone
        treatment were enrolled and received study treatment until disease progression. </textblock>
    </brief_summary>
  - <detailed_description>
      <textblock> For the study duration, all subjects maintained androgen deprivation with a gonadotropin releasing hormone (GnRH) agonist or
        antagonist or orchiectomy. Study drug was administered until disease progression. Disease progression was defined as a composite
        endpoint consisting of either clinical deterioration, radiographic progression or prostate-specific antigen (PSA) progression according to
        the Prostate Cancer Clinical Trials Working Group 2 (PCWG2) criteria. </textblock>
    </detailed_description>
    <overall_status>Completed</overall_status>
    <start_date type="Actual">July 9, 2012</start_date>
    <completion_date type="Actual">January 4, 2018</completion_date>
    <primary_completion_date type="Actual">January 4, 2018</primary_completion_date>
    <phase>Phase 2</phase>
    <study_type>Interventional</study_type>
    <has_expanded_access>No</has_expanded_access>
```


Figure 12. An example of a clinical trial report in XML format.

Feature Subcategory	Feature Name	Description/Definition
Class Label	Status	1 if Terminated, 0 if Completed
Administrative	Industry Collaborator	1 if Main Collaborator Class is industry 0 otherwise
	Number Collaborators	Number of listed collaborators from clinical trial XML
	Number Officials	Number of listed officials from clinical trial XML
	Responsible Party: Investigator	1 if Responsible Party is Investigator or Sponsor-Investigator
	Responsible Party: None Listed	1 if Responsible Party type is not listed
	Responsible Party: Sponsor	1 if Responsible Party is Sponsor or Sponsor-Investigator
	Industry Sponsor	1 if Sponsor class is industry, 0 otherwise
Eligibility	Average Eligibility Words	Average words per eligibility criteria
	Average Exclusion Words	Average words per exclusion criteria
	Average Inclusion Words	Average words per inclusion criteria
	Eligibility Lines	Number of eligibility criteria
	Eligibility Numbers	Number of numbers in eligibility
	Eligibility Words	Number of words in eligibility
	Exclusion Lines	Number of exclusion criteria
	Exclusion Numbers	Number of numbers in exclusion criteria
	Exclusion Words	Number of words in exclusion criteria
	Inclusion Lines	Number of inclusion criteria
	Inclusion Numbers	Number of numbers in inclusion criteria
	Inclusion Words	Number of words in inclusion criteria
	No Eligibility Requirement	1 if trial has no eligibility, 0 otherwise
	Gender Restriction	1 if trial has gender restriction, 0 otherwise
	Age Restriction	1 if trial has age restriction, 0 otherwise
	Healthy Volunteer	1 if trial accepts healthy volunteers, 0 otherwise
Study Design	Random Groups	1 if trial uses random groups, 0 otherwise
	Placebo Group	1 if trial has a placebo group, 0 otherwise
	Uses Blinding	1 if trial uses masking, 0 otherwise
	Number Arms	Number of groups
	Number Sites	Number of sites listed in clinical trial XML
Study Information	Has DMC	1 if trial has DMC, 0 otherwise
	Has Oversight	1 if trial has DMC or FDA regulation, 0 otherwise
	Has Expanded Access	1 if trial has expanded access, 0 otherwise
	FDA Regulation	1 if trial has FDA drug or FDA device regulation, o otherwise
	Main Country: USA	1 if main country is USA, 0 otherwise
	Number Countries	Number of countries listed in clinical trial XML
	No Phase	1 if no phase or phase 0, 0 otherwise
	Phase 1	1 if phase 1 or phase 1/2, 0 otherwise
	Phase 2	1 if phase 2 or phase 1/2 or phase 2/3, 0 otherwise
	Phase 3	1 if phase 3 or phase 2/3, 0 otherwise
	Phase 4	1 if phase 4, 0 otherwise
	Interventional Study	1 if trial is interventional, 0 if observational

Table 7. Summary of all 40 statistics features and their descriptions (definitions)

Clinical Trial XML Field	Description
study_design_info/allocation	Allocation of groups
sponsors/collaborator/agency_class	Class of collaborators
location_countries/country	Countries where the sites were located
detailed_description/textblock	Detailed description field
oversight_info/has_dmc	DMC committee
eligibility/criteria/textblock	Eligibility criteria
has_expanded_access	If the Clinical Trial has expanded access
oversight_info/is_fda_regulated_device	Studies a US FDA-regulated device product
oversight_info/is_fda_regulated_drug	Studies a US FDA-regulated drug product
eligibility/gender	Gender Restrictions
arm_group/arm_group_type	The role of each group in the clinical trial
intervention_browse/mesh_term	Intervention MeSH term
keyword	Keywords to describe description field
study_design_info/masking	Type of blinding used in trial
eligibility/maximum_age	Maximum age requirement
eligibility/minimum_age	Minimum age requirement
number_of_arms	Number of groups
overall_official/last_name	The officials of the clinical trial
location/facility	The sites in the clinical trial
phase	Phase of clinical trial
responsible_party/responsible_party_type	Responsible Party by official title
sponsors/lead_sponsor/agency_class	Class for main sponsor
overall_status	Recruitment Status
study_type	Observational or Interventional Study
eligibility/healthy_volunteers	If the study accepts healthy volunteers
condition_browse/mesh_term	Condition MeSH Term
start_date	Date clinical trial started

Table 8. Schema of XML fields used to extract features from clinical trial reports for analysis

References

1. Loper, E. & Bird, S. NLTK: The natural language toolkit. In *Proceedings of the ACL-02 Workshop on Effective Tools and Methodologies for Teaching Natural Language Processing and Computational Linguistics - Volume 1*, ETMTNLP '02, 63–70, DOI: 10.3115/1118108.1118117 (Association for Computational Linguistics, USA, 2002).
2. Pedregosa, F. *et al.* Scikit-learn: Machine learning in Python. *J. Mach. Learn. Res.* **12**, 2825–2830 (2011).
3. Mikolov, T., Chen, K., Corrado, G. & Dean, J. Efficient estimation of word representations in vector space. *CoRR* (2013).
4. Řehůřek, R. & Sojka, P. Software framework for topic modelling with large corpora. Available from: http://is.muni.cz/publication/884893/en (2010).
5. Robnik-Šikonja, M. & Kononenko, I. Theoretical and empirical analysis of ReliefF and RReliefF. *Mach. Learn.* **53**, 23–69, DOI: 10.1023/a:1025667309714 (2003).
6. Vergara, J. R. & Estévez, P. A. A review of feature selection methods based on mutual information. *Neural Comput. Appl.* **24**, 175–186, DOI: 10.1007/s00521-013-1368-0 (2013).
7. Lin, D. & Tang, X. *Conditional Infomax Learning: An Integrated Framework for Feature Extraction and Fusion*. ECCV'06 (Springer-Verlag, Berlin, Heidelberg, 2006).
8. Kraskov, A., Stögbauer, H. & Grassberger, P. Estimating mutual information. *Phys. Rev. E* **69**, DOI: 10.1103/physreve.69.066138 (2004).
9. Vergara, J. R. & Estévez, P. A. A review of feature selection methods based on mutual information. *Neural Comput. Appl.* **25**, 175–186 (2014).
10. Lecun, Y., Bengio, Y. & Hinton, G. Deep learning. *Nature* **521**, 436–444, DOI: 10.1038/nature14539 (2015).
11. Wang, S., Fu, L., Yao, J. & Li, Y. The application of deep learning in biomedical informatics. In *2018 International Conference on Robots Intelligent System (ICRIS)*, 391–394 (2018).
12. Kingma, D. P. & Ba, J. L. Adam: A method for stochastic optimization. *CoRR* **abs/1412.6980**, 1–15 (2015).
13. Ho, Y. & Wookey, S. The real-world-weight cross-entropy loss function: Modeling the costs of mislabeling. *IEEE Access* **8**, 4806–4813, DOI: 10.1109/access.2019.2962617 (2020).
14. Breiman, L. Random forests. *Mach. Learn.* **45**, 5–32, DOI: 10.1023/A:1010933404324 (2001).
15. Chen, T. & Guestrin, C. XGBoost: A scalable tree boosting system. In *Proc. of the 22nd ACM SIGKDD Conf. on Knowledge Discovery and Data Mining*, KDD '16, 785–794, DOI: 10.1145/2939672.2939785 (Association for Computing Machinery, New York, NY, USA, 2016).
16. Yu, H.-F., Huang, F.-L. & Lin, C.-J. Dual coordinate descent methods for logistic regression and maximum entropy models. *Mach. Learn.* **85**, 41–75, DOI: 10.1007/s10994-010-5221-8 (2011).
17. Davis, J. & Goadrich, M. The relationship between precision-recall and roc curves. In *Proc. of the 23rd Intl. Conf. on Machine Learning*, ICML '06, 233–240, DOI: 10.1145/1143844.1143874 (Association for Computing Machinery, New York, NY, USA, 2006).
18. Cortes, C. & Mohri, M. AUC optimization vs. error rate minimization. In *Proceedings of the 16th International Conference on Neural Information Processing Systems*, NIPS'03, 313–320 (MIT Press, Cambridge, MA, USA, 2003).
19. Ling, C., Huang, J. & Zhang, H. AUC: a statistically consistent and more discriminating measure than accuracy. *Proc. 18th Int'l Jt. Conf. Artif. Intell. (IJCAI)* (2003).
20. Chawla, N. V., Japkowicz, N. & Kotcz, A. Editorial: Special issue on learning from imbalanced data sets. *SIGKDD Explor. Newsl.* **6**, 1–6, DOI: 10.1145/1007730.1007733 (2004).
21. Demšar, J. Statistical comparisons of classifiers over multiple data sets. *J. Mach. Learn. Res.* **7**, 1–30 (2006).
22. Nadeau, C. & Bengio, Y. Inference for the generalization error. *Mach. Learn.* **52**, 239–281, DOI: 10.1023/A:1024068626366 (2003).
23. Bouckaert, R. R. & Frank, E. Evaluating the replicability of significance tests for comparing learning algorithms. In *Advances in Knowledge Discovery and Data Mining. PAKDD 2004*, vol. 3056, 3–12, DOI: https://doi.org/10.1007/978-3-540-24775-3_3 (Springer, 2004).
